# Transcriptome Analysis of the Central and Peripheral Nervous Systems of the Spider *Cupiennius salei* Reveals Multiple Putative Cys-Loop Ligand Gated Ion Channel Subunits and an Acetylcholine Binding Protein

**DOI:** 10.1371/journal.pone.0138068

**Published:** 2015-09-14

**Authors:** Päivi H. Torkkeli, Hongxia Liu, Andrew S. French

**Affiliations:** Department of Physiology and Biophysics, Dalhousie University, PO BOX 15000, Halifax, Nova Scotia, B3H 4R2, Canada; Sackler Medical School, Tel Aviv University, ISRAEL

## Abstract

Invertebrates possess a diverse collection of pentameric Cys-loop ligand gated ion channel (LGIC) receptors whose molecular structures, evolution and relationships to mammalian counterparts have been intensely investigated in several clinically and agriculturally important species. These receptors are targets for a variety of control agents that may also harm beneficial species. However, little is known about Cys-loop receptors in spiders, which are important natural predators of insects. We assembled *de novo* transcriptomes from the central and peripheral nervous systems of the Central American wandering spider *Cupiennius salei*, a model species for neurophysiological, behavioral and developmental studies. We found 15 Cys-loop receptor subunits that are expected to form anion or cation permeable channels, plus a putative acetylcholine binding protein (AChBP) that has only previously been reported in molluscs and one annelid. We used phylogenetic and sequence analysis to compare the spider subunits to homologous receptors in other species and predicted the 3D structures of each protein using the I-Tasser server. The quality of homology models improved with increasing sequence identity to the available high-resolution templates. We found that *C*. *salei* has orthologous γ-aminobutyric acid (GABA), GluCl, pHCl, HisCl and nAChα LGIC subunits to other arthropods, but some subgroups are specific to arachnids, or only to spiders. *C*. *salei* sequences were phylogenetically closest to gene fragments from the social spider, *Stegodyphus mimosarum*, indicating high conservation within the Araneomorphae suborder of spiders. *C*. *salei* sequences had similar ligand binding and transmembrane regions to other invertebrate and vertebrate LGICs. They also had motifs associated with high sensitivity to insecticides and antiparasitic agents such as fipronil, dieldrin and ivermectin. Development of truly selective control agents for pest species will require information about the molecular structure and pharmacology of Cys-loop receptors in beneficial species.

## Introduction

Pentameric Cys-loop receptors are ligand gated ion channels (LGIC) that mediate fast synaptic transmission in central and peripheral nervous systems. Many human diseases are caused by dysfunction of these channels, making them therapeutic targets for drugs [1]. Several invertebrate Cys-loop receptors are targets of widely used control agents, such as dieldrin, ivermectin and neonicotinoids [2–5]. Phylogenetic relationships, molecular structures and functions of these receptors have mainly been investigated in agricultural pests and parasites, rather than more beneficial arthropods. Several Cys-loop receptor genes have now been cloned from arachnids, mainly ticks and mites [6], but very little is known about these receptors in the largest arachnid order, the spiders [7]. Spiders are important natural predators of insects and may unintentionally be exposed to control agents. A recent report demonstrated that nicotinic acetylcholine (nACh) receptors of the pond wolf spider *Pardosa pseudoannulata* are activated by neonicotinoids [8], but Cys-loop subunit genes of other spider species have not been investigated.

Invertebrate Cys-loop receptors include anion permeable channels that are gated by γ-aminobutyric acid (GABA), glutamate (GluCl), histamine (HisCl) or protons (pHCl), and nematodes also have Cl-gated ACh and 5-HT receptors [9]. The only invertebrate cation permeable Cys-loop receptors are nicotinic acetylcholine channels (nACh) [6]. Pentameric receptors can be further divided into those containing only one conserved Cys-loop in their extracellular domain and those that have an additional disulfide bond that links the β9-β10 strands in Loop C. The glycine receptor, found only in chordates, is a prototype of receptors with two Cys-loops, and many invertebrate GluCl, HisCl and pHCl receptors are members of this group [10].

High-resolution structural information is useful for understanding how transmitter receptors function. Several Cys-loop receptor protein structures have recently become available with complete atomic resolution. These include the bacterial ligand-gated ion channels ELIC and GLIC [11,12], *C*. *elegans* GluCl channel [13,14], human GABA_A_β3 receptor [15] and mouse 5-HT_3_ receptor [16]. The only complete nACh receptor is a medium resolution electron microscopy structure from the electric ray *Torpedo marmorata* [17], but structural information for the channel extracellular domain has been inferred by homology with the molluscan acetylcholine binding protein AChBP [18]. All known Cys-loop receptors show remarkable structural similarities with conserved residues that are important for receptor function, making it possible to use a combination of several templates to infer information about other receptors to create homology models [19].

The Central American wandering spider, *Cupiennius salei*, is a widely used experimental animal, particularly for neurophysiological [20,21], and developmental studies [22,23]. Immunohistochemical and electron microscopic investigations have found a large number of GABA and glutamate containing neurons in the spider brain; some are efferents that project to the legs where they innervate mechanosensory neurons, and some project to muscles [24,25]. GABA or glutamate effects on the spider central neurons or muscle fibers have not been investigated, but electrophysiological experiments have revealed that agonists of Cys-loop GABA_A_ receptors (GABA and muscimol) and GluCl channels (glutamate and ibotenic acid) modulate the sensitivity of mechanosensory neurons of the lyriform VS-3 slit sensilla [26–29]. GABA_A_ agonists depolarize the neurons and induce brief inhibition followed by long lasting excitation [26,29] while GluCl agonists induce an identical response to GABA in some, but purely inhibitory response without any change in membrane potential in most VS-3 neurons [27,28]. Hence, there are probably multiple subtypes of Cys-loop receptors in each neuron.

The recently sequenced *C*. *salei* brain and hypodermis transcriptomes [30,31] provide an opportunity to investigate the genes that form Cys-loop receptors in the spider central and peripheral nervous systems. Here, we annotate sixteen genes that encode *C*. *salei* Cys-loop subunits and analyze their phylogenetic relationships and similarity with other species. To understand the structural and functional features of these molecules and predict which neurotransmitters and insecticides bind to each of them, we used sequence analysis and homology modelling with multiple templates and evaluated our results with Ramachandran plots.

## Materials and Methods

### Identification of Cys-loop ligand gated ion channel subunits from spider transcriptomes

Details of the *Cupiennius salei* transcriptome preparation, sequencing and assembly have been described previously [30]. Briefly, hypodermis membranes containing peripheral nerves and sensory neurons, glial cells and some muscle tissue, were collected from seven adult female spider siblings and two brains from different female spiders. The Qiagen RNeasy plus mini kit was used for hypodermis RNA extraction and a Qiagen RNeasy midi kit for brain RNA extraction. The mRNA separation, cDNA library construction and Illumina sequencing were performed by McGill University and Génome Québec Innovation Centre (Montréal, Québec, Canada). Initial cDNA reads (220 million pairs of 100 nucleotide length) were groomed by requiring at least 80 contiguous nucleotides with Phred score >19 to give a final database of ~110 million pairs of reads for each library.

Cys-loop LGIC subunit sequences were identified by searching the databases at low stringency against both nucleotide and amino acid sequences of published arthropod genes. Amino acid searches were conducted using BLOSUM45, BLOSUM62 and BLOSUM80 matching matrices [32]. All putatively matching reads were compared to the non-redundant protein database using BLASTX (http://blast.ncbi.nlm.nih.gov). Candidate reads were extended by the transcriptome walking algorithm [30] using an initial minimum overlap of 60 nucleotides. Minimum overlap was increased, in steps of 10 up to a maximum of 90 nucleotides, when required to eliminate multiple possible extension pathways in a few cases of highly redundant sequence structure. Overlap was reduced in steps of 10 to a minimum of 40 nucleotides when no overlaps were found in a complete pass through the database. Walking was always continued to exhaustion in the 5′ direction and continued to include the complete protein coding sequence (mORF or CDS) and the STOP codon in the 3′ direction. In many cases, the complete sequence was continued to the poly-A tail. Relative abundance of transcribed mRNA for each gene was estimated by counting matching Illumina reads to assembled genes as described earlier [31]. Data analysis and programming was performed in the C++ language using Microsoft Visual Studio and desktop computers. Multiple threaded code was used for searching and for walking, using up to 16 parallel processing cores.

### Phylogenetic analysis

Homologous sequences for the sixteen *C*. *salei* Cys-loop LGIC subunits (Table 1) found in the transcriptomes were individually searched using protein Delta-Blast. We selected representative examples of homologous invertebrate and vertebrate sequences for phylogenetic analysis. Sequence alignment was performed by the online version of the multiple sequence alignment software MAFFT Version 7 (http://mafft.cbrc.jp/alignment/server/) with default parameters (BLOSUM62, gap opening penalty 1.53) and G-Ins-I strategy with 200 SwissProt homologs and threshold E = 1e^-10^ [33]. We used the Guidance server (http://guidance.tau.ac.il/) to test the confidence of our alignments and removed unreliable sequences before further tests [34].

**Table 1:** *Cupiennius salei* Cys loop subunits.

**Putative Protein**	**Uniprot code**	**NCBI accession number**	**Number of amino acids**	**MW (kDa)**	**Clone identity to transcriptome**
CsGABArdl1	T1E1C3	GAKT01000019.1	504	57	F missing T /R SNP
CsGABArdl2	A0A061QHV7	GBFC01000040.1	478	54	F yes/R yes
CsGABA_A_β	T1DCD9	GAKT01000067.1	460	53	F yes/R yes
CsGABAgrd	T1E191	GAKT01000104.1	509	59	F yes
CsGluCl1	T1E1T2	GAKT01000005.1	399	46	F yes/F SNP
CsGluCl2	A0A061QFY0	GBFC01000041.1	401	47	F yes/R SNP
CsGluCl3	N/A	KT183364	411	47	N/A
CspHCl1	T1D1W5	GAKT01000068.1	468	54	R SNP
CspHCl2	T1D1Q6	GAKT01000128.1	469	53	F SNP/R SNP
CsHisCl1	A0A061QLK7	GBFC01000023.1	422	48	N/A
CsHisCl2	N/A	KT363688	365	43	N/A
CsNC-LGCl	A0A061QLN6	GBFC01000042.1	450	51	F yes/R yes
CsnACh1A	A0A061QLQ4	GBFC01000022.1	417	46	F yes/ F SNP
CsnACh1B	N/A	KT183361	417	48	R yes
CsnAChα	N/A	KT183362	539	62	N/A
CsAChBP	N/A	KT183363	246	29	N/A

GluCl = glutamate-gated chloride channel; GABA = γ-amino butyric acid; rdl = resistance to dieldrin; pHCl = pH gated chloride channel; grd = GABA and glycine-like receptor; nACh = nicotinic acetylcholine receptor; HisCl = histamine gated chloride channel; NC-LGCl = Not characterized ligand gated chloride channel; AChBP = acetylcholine binding protein. N/A = not available yet, submitted to GeneBank. F indicates forward and R reverse orientation in the clone direction. Sequences indicated with “yes” were identical to the transcriptome database and SNP indicates single nucleotide polymorphism. “missing T” means that a nucleotide with the nitrogenous base thymine was deleted.

After several rounds of pruning, re-aligning and testing, the final phylogenetic trees for the putative anion and cation channel subunits were created separately with MEGA 6 software using the Maximum likelihood method [35]. Based on the results from MEGA 6 “Models” the best protein model for the MAFFT aligned sequences using all sites was the Gamma distributed Le-Gascuel (LG + G) model for both groups [36]. Analysis of the putative cation channel group required addition of invariant sites (LG + G + I). The maximum likelihood analysis was performed with 1000 bootstraps using subtree pruning and extensive regrafting option with otherwise default settings. Accession codes and nomenclature for *C*. *salei* sequences are shown in Table 1 and sequences from other species that were used in the final analyses are listed in the S1 Table. The final trees were exported from MEGA6 as emf files and images created in Adobe illustrator CS5 (Adobe systems inc, San Jose, CA).

### Sequence analysis

The MAFFT aligned protein sequences were viewed and analyzed using Jalview 2.9. software (http://www.jalview.org/) [37]. For functional analysis, prediction of signal-peptide cleavage sites and membrane spanning regions we used the InterProScan 5 server (http://www.ebi.ac.uk/Tools/pfa/iprscan5/), which scans the sequence against the protein signatures of several databases [38]. Further sequence analysis was done with the MemPype server (http://mu2py.biocomp.unibo.it/mempype/default/index) [39].

Pairwise identity matrices were created using a Sequence demarcation tool with the MAFFT alignment and clustering the sequences with Neighbour joining tree [40]. The identity was also tested in Jalview by pairwise comparison and results from these two tests were closely similar.

### Protein structure prediction

We used the I-Tasser server to generate three-dimensional structure predictions of the *C*. *salei* Cys-loop subunits [41]. I-Tasser is a free user-friendly program that has been repeatedly ranked as the No 1 server in the Community Wide Experiments on the Critical Assessment of Techniques for Protein Structure Prediction (CASP). The sequences were uploaded in FASTA format and ran without restraints allowing the software to select the templates. Prior to the homology modelling, we removed the short N-terminal segments in front of the signal peptide cleavage sites that were found by InterPro scan for all other sequences except CsGABAgrd and CsGABAHisCl1 and 2 (S1 Fig and S2 Fig). These signal peptides are eliminated from mature proteins. We used the PyMOL Molecular Graphics System, Version 1.7.4 (Schrödinger, LLC.) software to generate images of the homology models. The stereochemical quality of predicted homology models were investigated by Ramachandran plot analysis using the RAMPAGE server [42].

### PCR analysis and cDNA cloning

Total RNA was extracted from the leg hypodermal tissues using a Qiagen RNeasy plus mini kit as described previously [31]. Reverse transcription (RT) was performed using 200 ng total RNA and oligo d(T)23VN primer with ProtoScript II reverse transcriptase (New England BioLabs, Whitby, ON, Canada). The synthesized cDNA was used as a template to amplify the Open Reading Frame (ORF) regions of the genes using gene specific primers with designs based on the assembled sequences using Q5 High-Fidelity DNA Polymerase (New England BioLabs). The PCR product size was verified by running with a DNA marker on an agarose gel and then gel-purified using GenElute Gel Extraction Kit (Sigma Aldrich, Oakville, ON, Canada). A-tailing reaction was then performed by incubating the purified PCR product with dATP and Taq DNA polymerase (New England BioLabs). After the tailing reaction, the DNA was used for ligation with pGEM®-T Easy Vector and then transformed into JM109 competent cells (Promega, Madison, WI). After verifying by colony PCR, the positive clones were selected for plasmid purification using GenElute Plasmid Miniprep kit (Sigma Aldrich) and sequenced by the McGill University and Génome Québec Innovation Centre.

## Results and Discussion

### 1. Cupiennius salei brain and hypodermis transcriptomes have sixteen putative Cys-loop ligand gated ion channel subunits

All Cys-loop LGICs are composed from a ring of five subunits that have an extracellular ligand binding domain made mainly from β-sheets and an α-helical transmembrane domain [13,15,16]. Deduced proteins from sixteen mRNA sequences in *C*. *salei* brain and hypodermis transcriptomes (Table 1, S1 Fig and S2 Fig) had conserved features of Cys-loop LGIC subunits. They had extracellular N-terminal regions with the characteristic Loops A to G that form the ligand-binding site and a cysteine loop in the extracellular domain. Five subunits also had a disulphide bond in the Loop C. This feature is similar to the vertebrate glycine receptor and invertebrate GluCl channels [10]. Most sequences had the typical four membrane spanning regions (TM1-TM4) and a highly variable loop between TM3 and TM4 that has been shown to associate with cytoskeletal proteins such as gephyrin or rapsyn, and is believed to contribute to channel kinetics [43,44]. However, one subunit, the CsAChBP lacked the transmembrane regions completely.

We analyzed twelve *C*. *salei* Cys-loop gene sequences by PCR and cloned them. In most cases cloning produced identical sequences to the transcriptome database (Table 1). Some cloned sequences had single nucleotide polymorphisms (SNP) that encoded the same amino acids and were present, but less well represented in the transcriptomes than the canonical sequence. The only gene that could not be amplified by PCR was *CsHisCl1*.

InterPro scan identified all 16 proteins from the transcriptomes as subunits of neurotransmitter gated ion channels. Based on conserved motifs in transmembrane regions, twelve subunits were predicted to form pentameric anion permeable channels. The remaining four subunits had typical ligand binding motifs for cation channels in the nicotinic acetylcholine receptor (nACh) family. CsnAChBP did not have TM regions, but the other three subunits had TM motifs that were also typical for nACh channels. Various InterPro databases provided evidence for further classification of some subunits. We have combined this information with the evidence from phylogenetic and sequence analysis (below) to classify the sequences as shown in Table 1. Complete *C*. *salei* Cys-loop amino acid sequences are aligned with homologous sequences in S1 Fig and S2 Fig.

### 2. All sixteen Cys-loop genes are present in the *C*. *salei* brain and leg hypodermis

**Fig 1:**
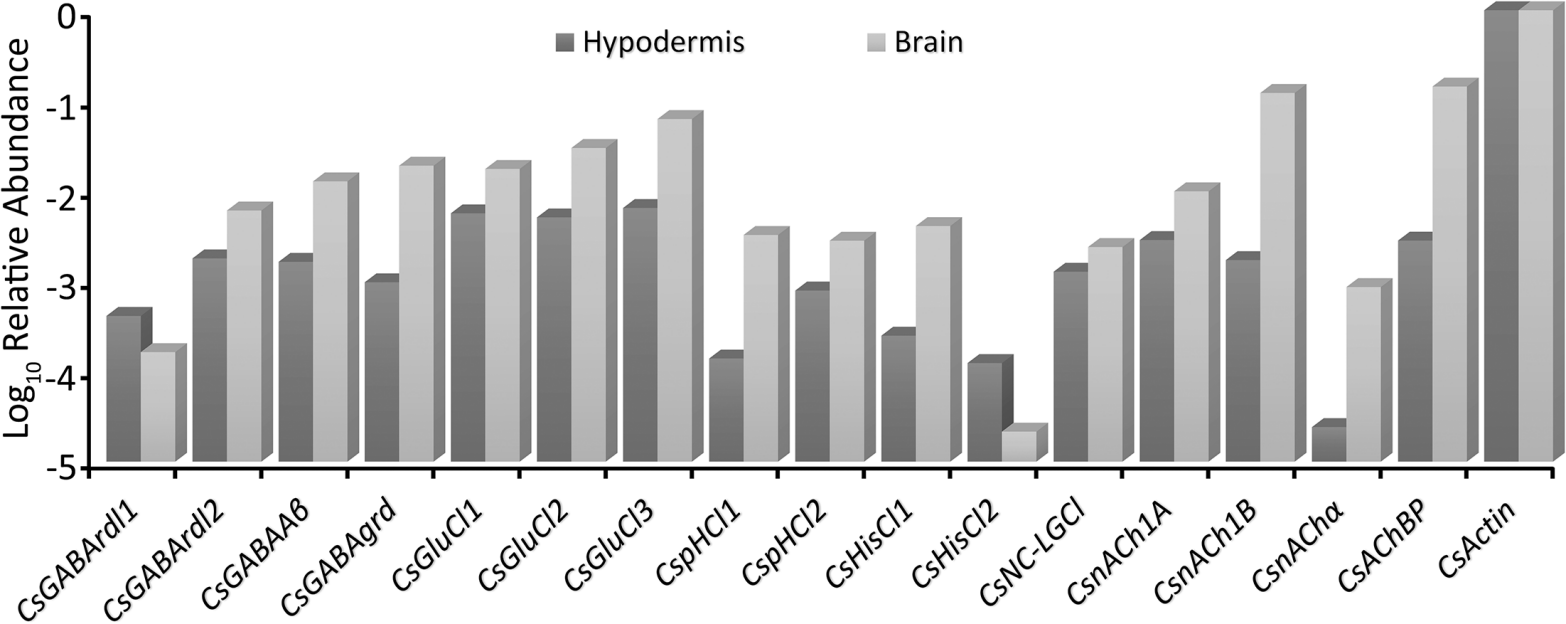
Cys-loop subunit abundances. Relative abundances of Cys-loop receptor subunits in *C*. *salei* brain and hypodermis transcriptomes compared to the abundance of actin. The data were obtained by counting total reads in the transcriptome libraries with at least 90 consecutive identical nucleotides to the reading frame of each gene, then normalizing by reading frame length. The scale is logarithmic. Accession numbers and sequence nomenclature are in Table 1.

We estimated the relative abundance of transcribed mRNA for each Cys-loop sequence by searching the groomed transcriptome data for matches to the main open reading frame (mORF) of each gene [31]. Depending on their abundances, we tested 40–230 million reads for each gene requiring a minimum of 90/100 identical nucleotide matches to score each read as derived from the gene. We normalized the total counts by mORF length, and then expressed them as abundance relative to the putative gene that encodes actin. *Actin* RNA accounted for about 0.4% of all reads [31]. Fig 1 shows the relative abundances of each *C*. *salei* Cys-loop gene in the brain and hypodermis transcriptomes. We found all 16 sequences in both tissues, but in most cases, the relative abundance was higher in brain than in hypodermis. Exceptions were the *CsGABArdl1* and the *CsHisCl2* whose relative abundances were higher in the hypodermis than in the brain. The nACh sequences were generally much sparser in hypodermis than in the brain, and the *CsnAChα* had a significantly lower abundance in the hypodermis than any other gene.

Both the brain and hypodermis tissue that we used for the transcriptome preparation contain mainly neurons and glial cells, and most of the Cys-loop subunits are likely to originate from neurons. However, some muscle tissue was attached to the hypodermis and it is possible that some of the Cys-loop subunits originate from leg muscle. Arthropod skeletal muscles are innervated by glutamatergic motor neurons [45] and excitatory glutamate receptors that are not Cys-loop receptors are likely to mediate muscle contraction. However, nerve fibers containing GABA also innervate *C*. *salei* and other arthropod muscle fibers (45, 25). Thus, Cys-loop receptor subunits are also likely to be present in muscle.

### 3. Molecular phylogenetic analysis found members of *C*. *salei* Cys-loop superfamily in eight major clades

**Fig 2:**
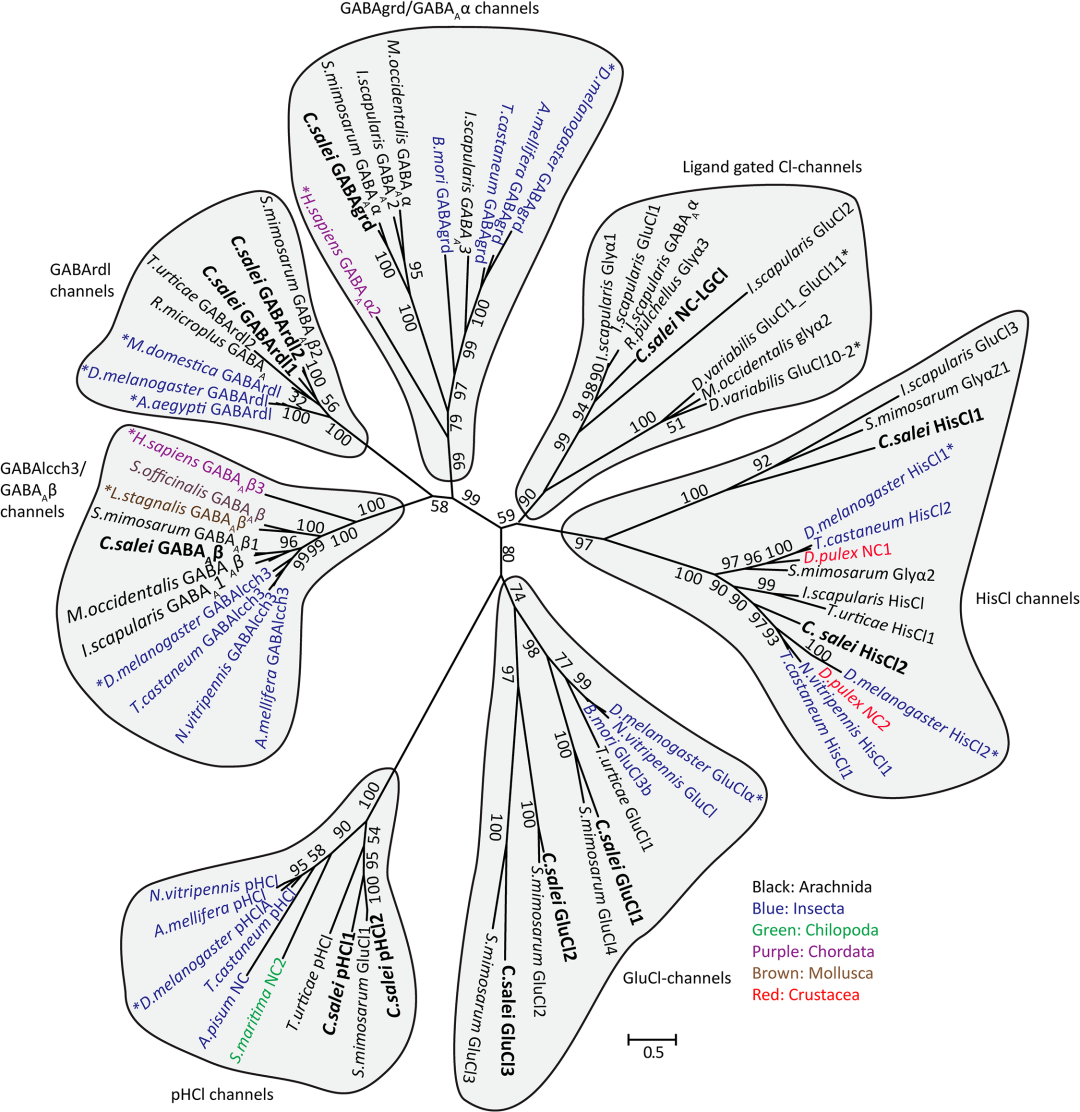
Molecular phylogenetic analysis of putative anion permeable channel subunits. Evolutionary history inferred by the Maximum Likelihood method. The unrooted radiation tree is drawn to scale, with branch lengths measured in numbers of substitutions per site. The analysis involved 72 MAFFT aligned amino acid sequences and was run with 1000 bootstraps. Numbers indicate bootstrap values. There were a total of 1385 positions in the final dataset. Evolutionary analyses were conducted in MEGA6 [35]. Accession numbers and nomenclature for *C*. *salei* sequences are in Table 1 and for other sequences in S1 Table.

**Fig 3:**
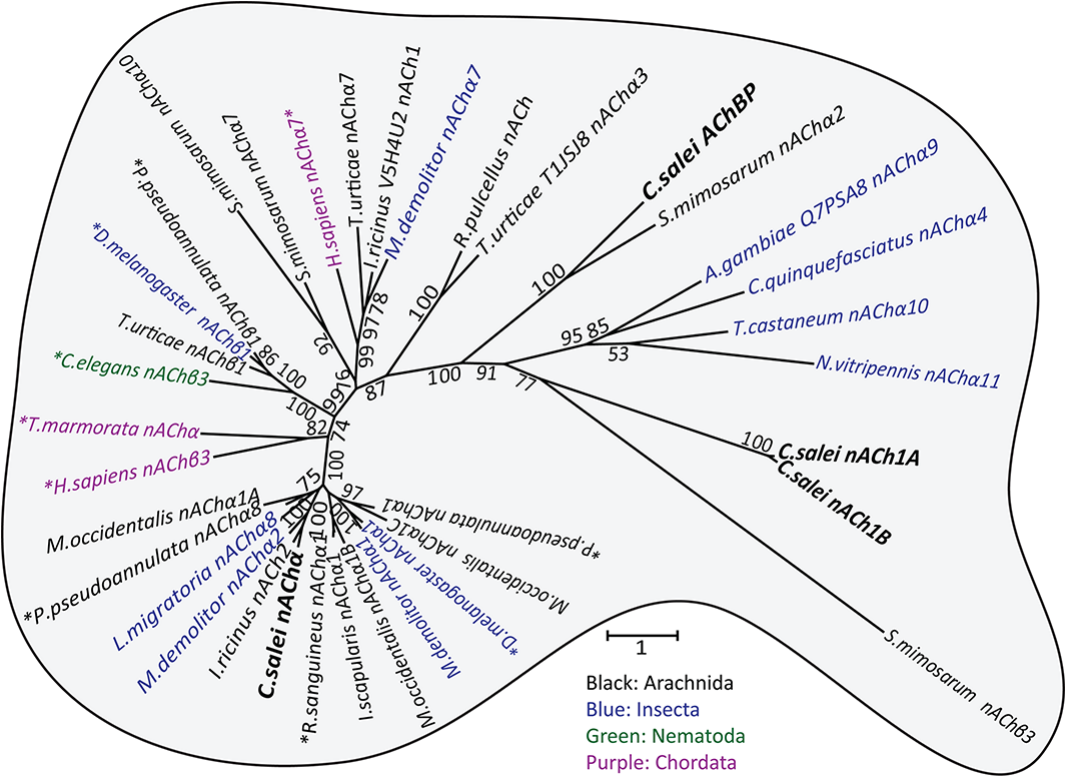
Molecular phylogenetic analysis of putative nACh channel subunits. Evolutionary history inferred by the Maximum Likelihood method. The unrooted radiation tree is drawn to scale, with branch lengths measured in numbers of substitutions per site. The analysis involved 36 amino acid sequences and was run with 1000 bootstraps. Numbers indicate bootstrap values. There were a total of 903 positions in the final dataset. Evolutionary analyses were conducted in MEGA6 [35]. Accession numbers for *C*. *salei* sequences are in Table 1 and for other sequences in S1 Table.

**Fig 4:**
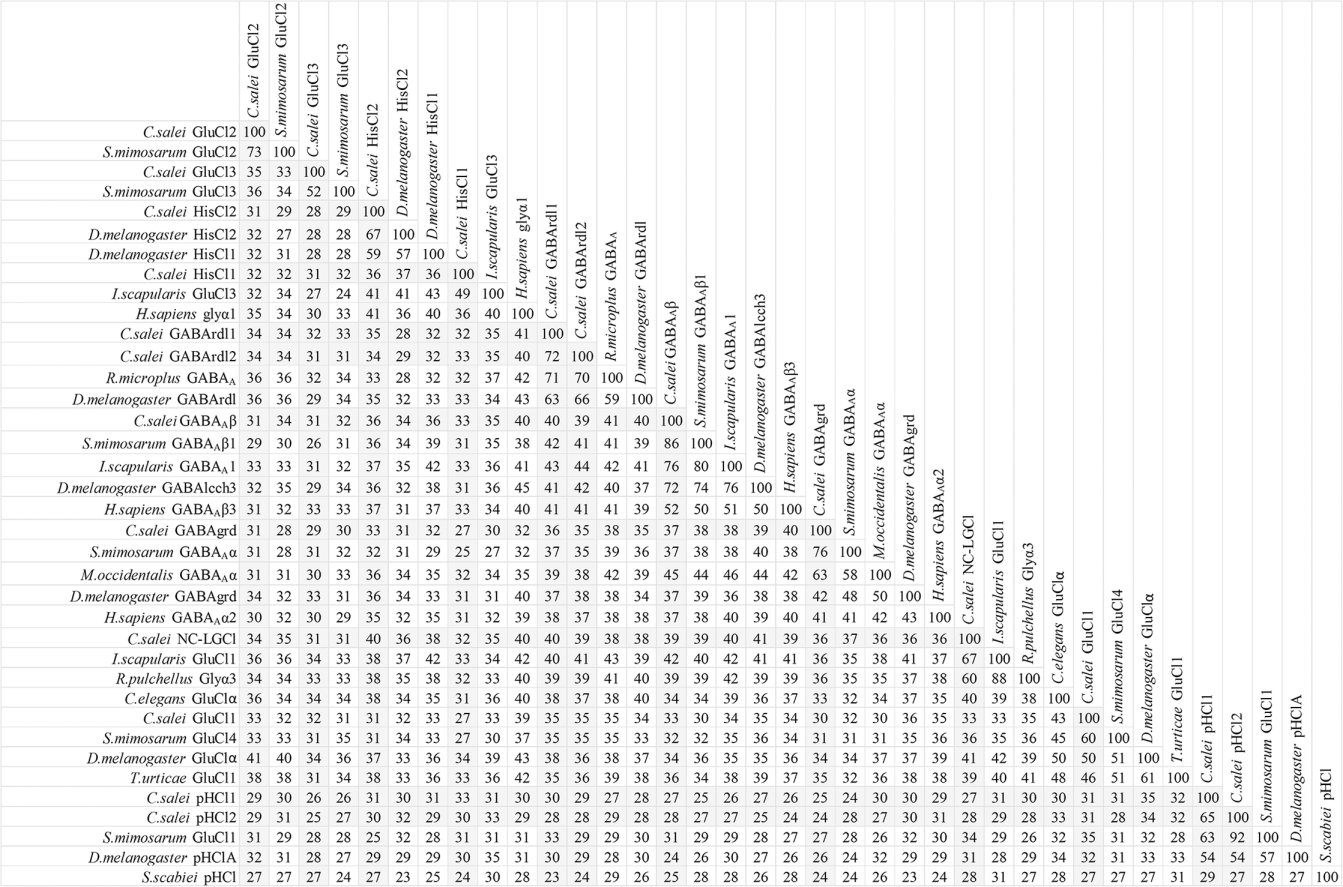
Pairwise identity matrix for anion permeable Cys-loop sequences. Each cell represents a percentage identity score between two sequences, one indicated horizontally to the left and the other vertically at the top. The 11 *C*. *salei* sequences are in bold and their scores are shaded in grey. The sequence demarcation tool for MAFFT aligned sequences [40] was used to create the matrix.

**Fig 5:**
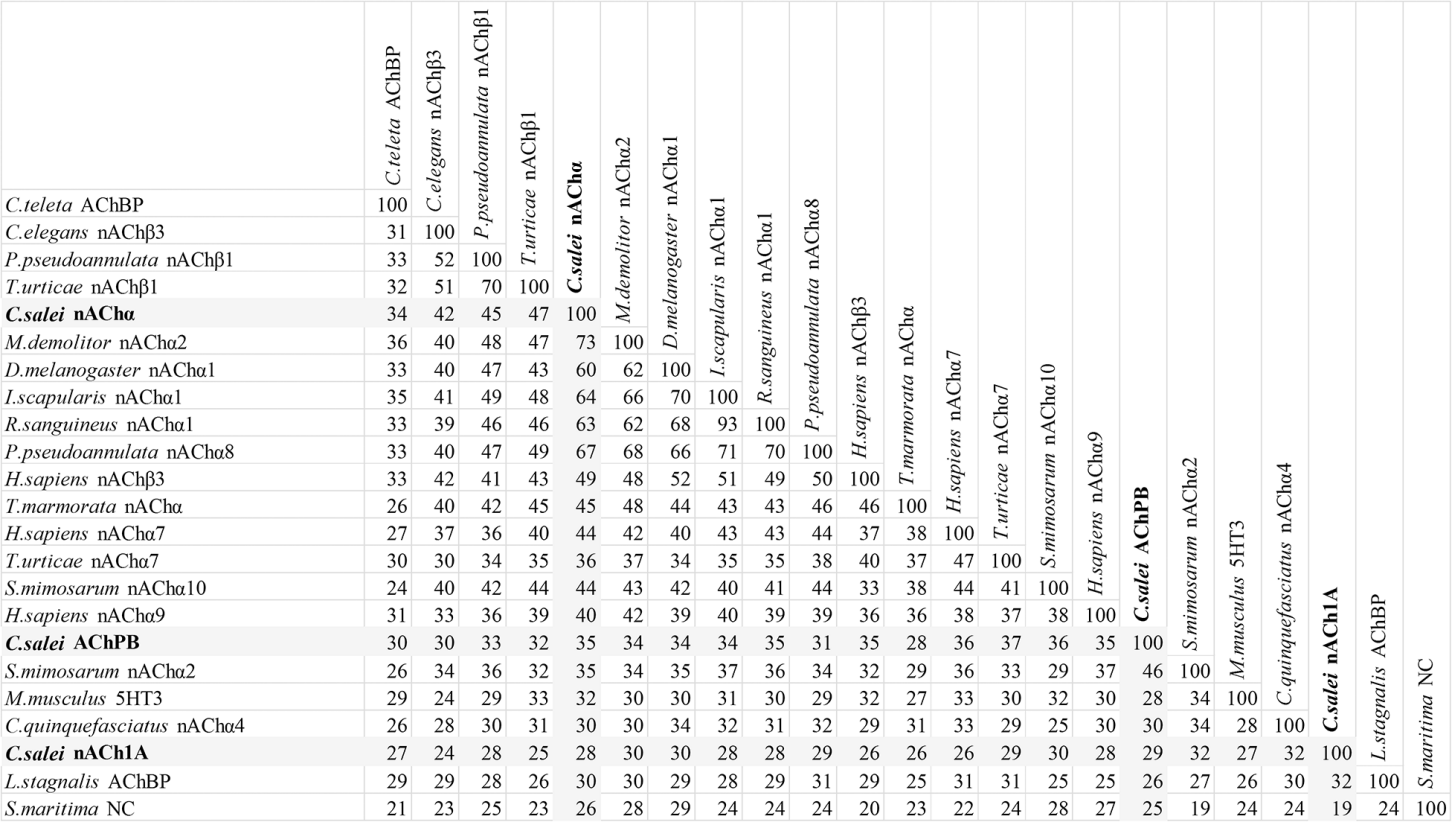
Pairwise percentage identity score matrix for cation selective Cys-loop sequences. Each cell shows the score between two sequences, one indicated horizontally to the left and the other vertically on the top. Three *C*. *salei* sequences are in bold and their scores are shaded in grey. The sequence demarcation tool for MAFFT aligned sequences [40] was used to create the matrix.

To clarify the evolutionary relationships of the *C*. *salei* Cys-loop subunits to homologous sequences in other species we performed molecular phylogenetic analysis based on the Maximum likelihood algorithm. *C*. *salei* Cys-loop sequences clustered with high bootstrap values into eight orthologous clades found in several invertebrate phyla with the closest homologs in other arachnid species. Some are also orthologous to mammalian and other vertebrate Cys-loop receptors. One radiation tree (Fig 2) includes representative examples of the seven clades in the anion selective channel group, while another (Fig 3) shows the nACh receptors. Alternative phylogenetic analyses based on the Neighbour-joining method did not reveal significant differences in the topology of either tree. Figs 4 and 5 show pairwise identity matrices for most of the same sequences as the phylogenetic trees. These figures also include a small number of sequences that were not included into the final trees, but whose 3-dimensional structures and/or physiological functions have been characterized and are discussed below.

The phylogenetic analyses indicated that *C*. *salei* transcriptomes contained RNA that encode most of the same types of Cys-loop receptors that have been described in other invertebrate species, although some sequences appear to be more specific to arachnids or spiders. In addition, we found a putative ACh binding protein (AChBP) that has only previously been described in molluscs and in one annelid [46,47]. Inclusion of *C*. *elegans* Cys-loop sequences into analysis produced trees with low bootstrap values, so we excluded them from the final analyses. However, we tested the phylogenetic relationships of spider and *C*. *elegans* sequences separately and found orthologous groups, except for the nematode Cl-gated ACh or 5-HT receptors [9].

Most invertebrate sequences included in our analyses have previously been annotated based on homology, not actual function. Sequences that have been functionally tested by expression in heterologous systems and/or by agonist binding studies are indicated by asterisks in Figs 2 and 3 and noted in the S1 Table. Some invertebrate sequences have been annotated as glycine receptors by homology, but we do not know of any evidence that these sequences are glycine receptors, which are otherwise found exclusively in chordates [10].

#### 3.1. GABA_A_ receptor subunits

Of the four putative GABA_A_ receptor subunits (Fig 2) two are in the invertebrate “resistance to dieldrin’ (rdl) clade that has no vertebrate orthologues, one is in the “GABA and glycine-like receptor” (grd) clade that is orthologous to vertebrate GABA_A_α subunits, and one is in the GABA_A_β clade that is a highly conserved clade in vertebrates and invertebrates. This clade is named “ligand gated chloride ion channel homolog 3”, lcch3 in insects [48]. Mammalian pentameric GABA_A_ receptors typically have two α, two β and one γ subunit, and GABA binding sites are located between the α-β interface [49]. Invertebrate GABA_A_ receptor subunit composition is not known, but expression of insect *rdl* genes in oocytes produced functional homopentameric receptors that had similar properties to their native GABA receptors; they were Cl-channels that could be blocked by picrotoxin, but not by bicuculline [50–52]. Conversely, co-expression of *Drosophila rdl* and *lcch3* genes formed channels that had different properties to native receptors [53]. Expression of neither *Drosophila lcch3* nor *grd* gene on their own produced functional channels, but when co-expressed in *Xenopus* oocytes, they formed GABA-gated channels that were selective to cations [54]. However, receptors with similar properties have not been found in native insect tissue. The mechanosensory VS-3 neurons in *C*. *salei* leg patella respond to GABA with depolarization and this response is blocked by picrotoxin but not by bicuculline [26], suggesting that these cells have GABArdl receptors. Whether the GABA_A_β and grd are also subunits of these receptors, or have other roles in spider tissue remains to be investigated.

Insects have one GABA*rdl* gene that encodes several alternatively spliced subunits with different agonist sensitivities [55]. They also have one *lcch3* and one *grd* gene [56–59]. The spider mite, *T*. *urticae*, has three *rdl* genes but no β or *grd* genes [6]. Multiple *rdl* genes are also common in other arachnid species, and some appear to lack genes that encode other GABA_A_ subunits [6]. However, we found arachnid GABA subunits in all three clades (Fig 2). For example, the African social velvet spider *Stegodyphus mimosarum* has a homologous gene for each of the *C*. *salei* GABA_A_ sequences, but only one *rdl* gene. Therefore, the numbers of different GABA_A_ sequences vary even in closely related arachnid species.

#### 3.2. Glutamate-Gated Chloride Channel (GluCl) subunits

We found three putative GluCl genes (*CsGluCl1-3*) in the *C*. *salei* transcriptomes. Each had a homologous counterpart in the *S*. *mimosarum* genome (Fig 2). Most insects have only one GluCl subunit [57,58] and these are phylogenetically closest to the CsGluCl1. The spider mite *T*. *urticae* has six orthologous GluCl subunits [6] and although only one is shown in Fig 2, they were all in the same sub-clade with the CsGluCl1, more distant to the other two CsGluCl subunits. We also tested six *C*. *elegans* GluCl sequences separately [9] and found that they are also more closely related to the CsGluCl1 than to the other two *C*. *salei* GluCl subunits. The above-mentioned *S*. *mimoasarum* sequences where the only closely homologous sequences to CsGluCl2 and CsGluCl3, suggesting that these sub-clades may be specific to spiders. Similar to the insect and *T*. *urticae* 12344 Cl-channel clade, the CsGluCl2 and CsGluCl3 sequences lack the second Cys-loop that is usually found in GluCl channels [6]. However, the *C*. *salei* transcriptomes did not have any sequences in the 12344 clade and it was not included in our phylogenetic tree.

Insect and *Aplysia* GluClα subunits formed homomultimeric glutamate gated Cl-channels that were activated irreversibly by ivermectin in expression systems [10,52,60]. However, a functional *C*. *elegans* GluCl receptor required both α and β subunits [13,61]. *Drosophila* and *Musca domestica* GluCl subunits were shown to co-assemble with GABArdl subunits and the latter formed heteromultimeric channels with higher GABA sensitivity than the homomultimeric GABArdl channel [52,62]. Arachnid GluCl or GABA channels have not been tested in expression systems. Spider VS-3 neurons had variable responses to glutamate in electrophysiological experiments [27,28]: Some cells responded to both glutamate and GABA with a large depolarization and brief inhibition followed by excitation. However, glutamate did not depolarize most neurons at all or only slightly, but inhibited responses to mechanical and electrical stimulation. These differences suggest a heterogeneous population of GluCl channels in different VS-3 neurons with varying agonist sensitivities and ionic selectivities.

#### 3.3. pH gated chloride channels (pHCl)

Two *C*. *salei* subunits are in the pHCl clade that includes representatives from many insect and arachnid species [6,58]. The pH sensitivity of these channels was demonstrated in *Drosophila* [63] and itch mite *Sarcoptes scabiei* [64] pHCl subunits expressed in oocytes. Both subunits formed homopentameric Cl-permeable channels that were insensitive to Cys-loop receptor agonists including GABA, glutamate and ACh, but alkaline pH induced inward currents. Both channels were also sensitive to ivermectin, which produced a slowly deactivating or irreversible inward current that was larger at alkaline pH [63,64]. The *Drosophila* pHCl sequence shares 54% identity with both *C*. *salei* pHCl subunits, but the mite pHCl sequence is less similar to *C*. *salei* pHCl, or any insect pHCl sequences (Fig 4). Furthermore, we were not able to obtain reliable results with phylogenetic analysis when we added the *S*. *scabiei* subunit to our database. The structural features that provides pH sensitivity to the *Drosophila* or *S*. *scabiei* channels are unknown.

#### 3.4. Histamine-gated Cl-channels (HisCl)

Two *C*. *salei* subunits are in the HisCl-channel clade. The CsHisCl1 subunit is in a distant branch to most insect, arachnid and crustacean HisCl subunits. It forms a separate branch with arachnid subunits that have been annotated as glycine or glutamate gated Cl-channels (Fig 2). Since none of these channels has been functionally tested, we do not know which transmitter(s) activate them, or their functional roles. The CsHisCl2 is orthologous to the insect HisCl channel that has been shown to mediate neurotransmission in *Drosophila* eye where histamine activates HisCl2 (also called hclA or ort) channels in the second order lamina neurons [65,66]. *Drosophila* HisCl1 (also called hclB) subunits also formed histamine gated Cl-channels in expression systems, but had different properties than the native receptors and were only found in laminar glial cells [66,67]. Our phylogenetic analysis found orthologous sequences to CsHisCl2 subunits in several arachnid species, including the spider *S*. *mimosarum*. Our transcriptomes did not originate from spider eyes, indicating that CsHisCl2 is not exclusively expressed in the eye. However, *CsHisCl2* RNA abundance was very low in the brain and higher in the leg hypodermis (Fig 1), which has a large number of sensory neurons.

#### 3.5. Non-Characterized Ligand Gated Chloride Channels (NC-LGCl)

One of the *C*. *salei* sequences is in a distinct group of putative ligand gated Cl-channels that has only arachnid members. Arachnid orthologues to this sequence have been named either GABA, glycine or GluCl subunits, but there is no experimental evidence that any of these transmitters gate these channels. Therefore, we named the *C*. *salei* sequence NC-LGCl. The most closely related functionally expressed sequences are the American dog tick, *Dermacentor variabilis*, GluCl10-2, GluCl1 and GluCl11 (US patents 7267964 and EP2009021). Homomultimeric GluCl10-2 expressed in *Xenopus* oocytes were not sensitive to glutamate or ivermectin, while homomultimeric GluCl1 and 11 formed Cl-channels that were activated by both glutamate and ivermectin.

#### 3.6. Nicotinic Acetylcholine Receptor Subunits (nACh)

Compared to other invertebrate nACh receptor subunits our *C*. *salei* transcriptome searches revealed relatively few nACh subunits. Most insect genomes have 10–16 nACh subunits, while the *T*. *urticae* genome had 10 [6]. It is likely that spider has more nACh subunits in tissues other than the brain and hypodermis. Classification of nACh receptor subunits is complicated by differences in nomenclature among different species. Generally, α-subunit Loop C has two vicinal cysteines that are necessary for agonist binding, while non-α subunits lack the vicinal cysteines and are sometimes classified as β subunits [68,69]. One of the *C*. *salei* nACh proteins was orthologous to nAChα subunits (Fig 3). Closely related proteins include sequences from the wolf spider *Pardosa pseudoannulata* and the tick *Rhipicephalus sanguineus* that share 67% and 63% similarity with CsnAChα (Fig 5). Both of these sequences have been expressed in *Xenopus* oocytes, but similarly to most invertebrate nACh subunits, they failed to function as homomultimers [8,70]. When co-expressed with nAChβ subunits, they formed functional ACh and nicotine sensitive receptors.

The spider transcriptomes had two non-α subunit isomers, CsnACh1A and B that lacked the vicinal Loop C cysteines. Phylogenetically these sequences formed a different branch to other nACh sequences that was closest to a branch containing only a *S*. *mimosarum* subunit named nAChβ3. Exhaustive GeneBank search did not find any closer homologues to these subunits than those shown in Fig 3. It is not clear what the functions of these subunits are, whether they form heteromultimeric channels with the CsnAChα or some other subunits. Since the mRNA level of these non-α subunits are higher in the brain and hypodermis than the level of α subunits (Fig 1) they are likely to have an important role in regulating the functions of both central and sensory neurons.

#### 3.7. Acetylcholine Binding Protein (AChBP)

CsAChBP shares a branch of the phylogenetic tree with the *S*. *mimosarum* fragment that has been named nAChα2, but may be an ACh binding protein. Since AChBPs have previously only been found in molluscan and annelid species, we ran additional phylogenetic analysis by adding AChBP sequences to the nACh group and a separate analysis that only included AChBPs, but these tests produced trees with low bootstrap values. This is probably because AChBP sequence conservation is low, even within molluscan species [46]. Although several AChBPs have been shown to bind ACh and nicotine, their physiological roles remain unclear [18,47,71]. Molluscan glial cells secrete AChBPs that are believed to regulate synaptic transmission by buffering ACh levels in the synapses instead of, or in addition to, acetylcholine esterase (AChE) [46]. AChE is widely found in metazoans and is important in regulating synaptic transmission by reducing ACh levels [47]. We also found four AChE sequences in the *C*. *salei* transcriptomes, making it unlikely that AChBP would be needed to regulate ACh levels in spider tissue.

### 4. Sequence analysis

**Fig 6:**
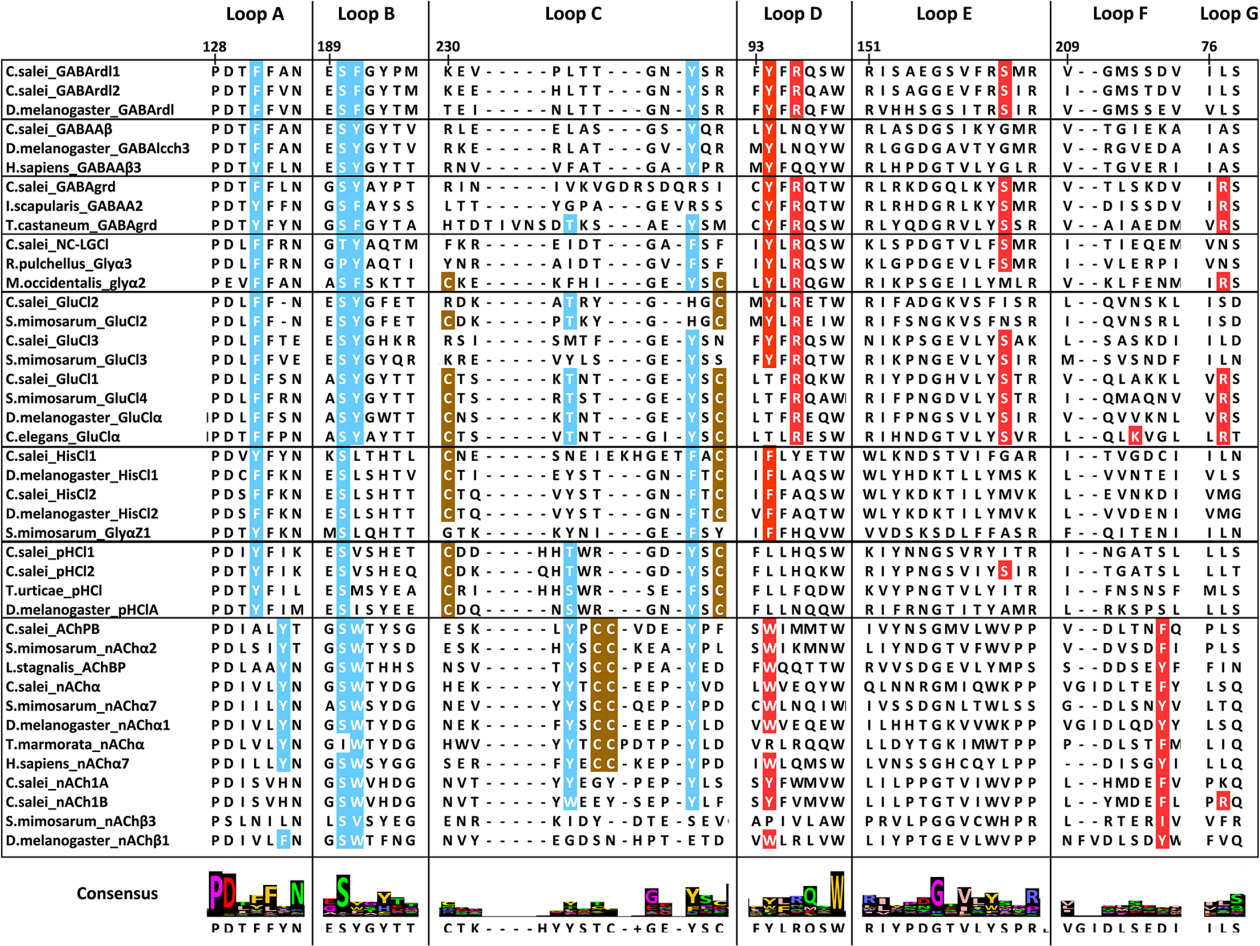
Alignment of the amino acid sequences in the N-terminal agonist binding Loops. Conserved residues involved in agonist binding to the principal face are highlighted in blue and those to the complementary face are shown in red. Cysteine residues of the second Cys-loop in GluCl, HisCl and pHCl subunits are indicated in brown and the vicinal cysteine residues in the Loop C of the nAChα subunits are also shown in brown. Numbering is shown for the *C*. *salei* GABArdl1 subunit. Sequences were aligned using MAFFT.

**Fig 7:**
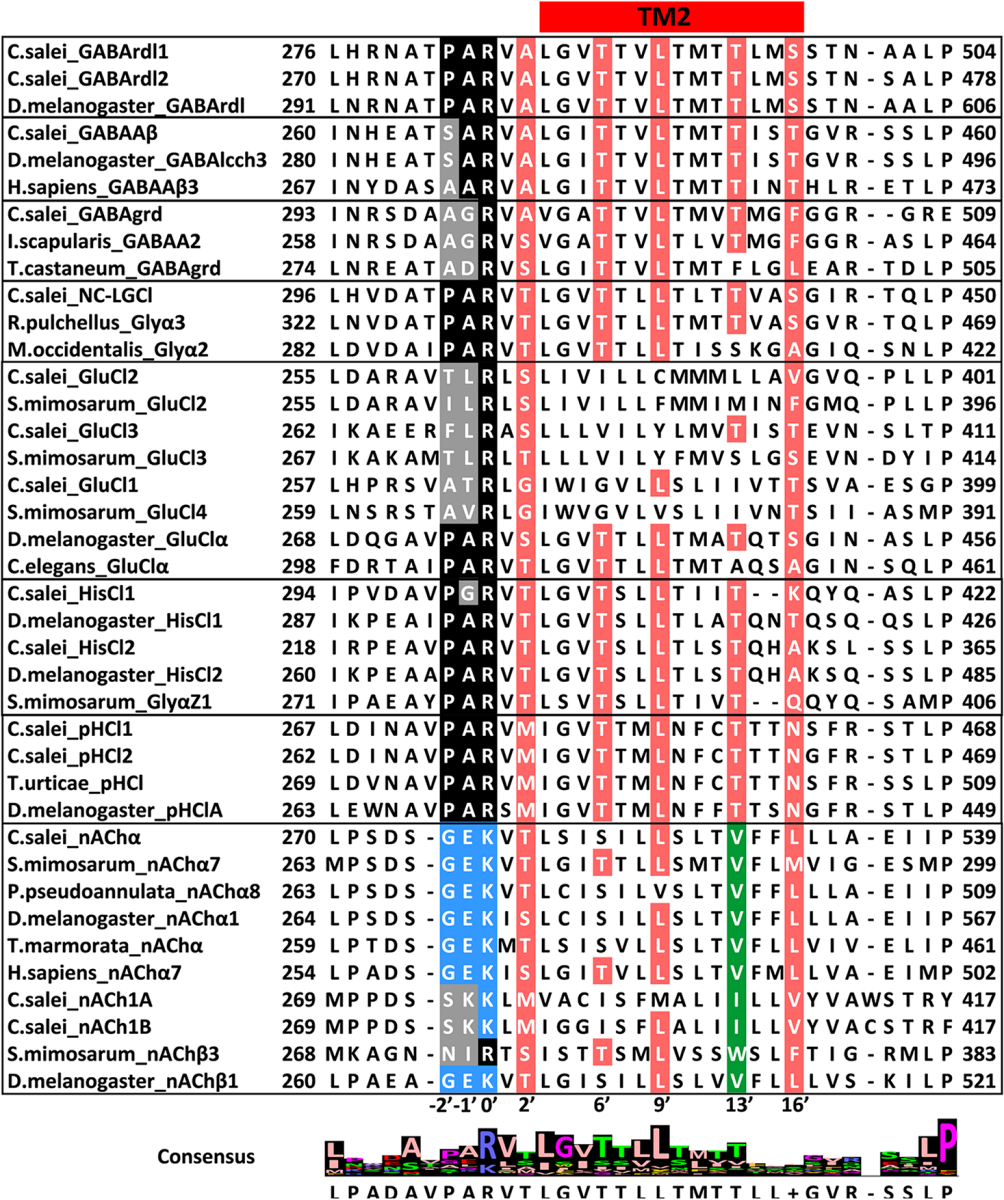
Alignment of the amino acid sequences in transmembrane domain 2 (TM2). Residues that line the pore in most Cys-loop receptors are shown in red. Arginine in position 0’ is critical for Cl-selective channels. The equivalent position for cation selective channels (-1’) generally has negatively charged residue (E or D). Note that the two isomers of *C*. *salei* nACh1 have a positively charged amino acid in this location. Position 13’, which is occupied by hydrophobic residues in cation permeable channels, is shown in green. Sequences were aligned with MAFFT.

Functional Cys-loop receptors consist of five subunits. Agonists bind to a specific binding pocket region on the large extracellular N-terminal domains of two neighbouring subunits. It is formed by three loops A–C of the principal face that span β strands and contain key aromatic residues. The adjacent subunit, which forms the complementary face, contributes three to four β strands with residues clustered in segments called loops D–F [18,44,72,73]. The binding sites of all Cys-loop receptors are dominated by aromatic residues but the critical residues are not necessarily equivalent even for closely related receptors [74–76]. Each subunit has four transmembrane helices TM1-TM4. The TM2 helix is located centrally and forms the channel pore; its amino acid sequence determines ion selectivity [43]. Portions of the *C*. *salei* sequences aligned with representative homologous sequences from other species containing the agonist binding loops and the TM2 are shown in Figs 6 and 7, respectively. The complete sequence alignments are in S1 Fig and S2 Fig.

#### 4.1. Extracellular ligand binding domain in *C*. *salei* Cys-loop subunits

All twelve subunits in the anion permeable channel family and the CsnAChα subunit have well-conserved Cys-loops with 13 amino acids between the cysteine residues (S1 Fig and S2 Fig) [1]. The CsnACh1A and 1B subunits have 14 residues in their Cys-loops, which is unusual. CsAChBP has the most atypical Cys-loop with 16 amino acids. AChBPs have variable numbers of residues in their Cys-loops, even in different molluscan species [18,77]. Since AChBPs do not have the channel region, the Cys-loop must have a different role than in “normal” receptors where it is involved in communication between the neurotransmitter binding site and the pore [73]. The second Cys-loop, which is commonly found in glycine and GluCl receptors, occurs only in one of the *C*. *salei* GluCl subunits (CsGluCl1), but it is present in CsHisCl1 and 2 as well as CspHCl1 and 2 subunits (Fig 6, S1 Fig).

GABA binds to a range of vertebrate and invertebrate Cys-loop receptors, but the actual binding site varies [75]. The CsGABArdl1 and 2, the CsNS-LGIC and CsGluCl3 are the only *C*. *salei* anion channel sequences that have all the residues believed to be involved in agonist binding in both the principal face (blue residues in Fig 6) and the complementary face (red residues in Fig 6). The CsGABA_A_β and CsGluCl1 subunits have all of these residues only on the principal face and lack some of them on the complementary face while the CsGABAgrd subunit has all the same complementary face amino acids involved in agonist binding, but lacks one on the principal face. All other putative anion selective subunits lack some or all of these residues. This arrangement suggests that, at least in some of the pentameric channels, different subunits form the principal and complementary faces for agonist binding. Since activation of Cys-loop receptors generally requires binding of at least two agonist molecules [78], additional subunits may have different roles.

In addition to the aromatic ring required for agonist binding in all Cys-loop receptors, arthropod and nematode GluCl channel glutamate recognition was recently shown to depend on an arginine residue in Loop G [79]. In *C*. *salei*, this arginine is only present in the CsGluCl1 subunit (Fig 6), but not in the other two GluCl subunits. Interestingly, along with all other complementary face agonist binding residues, this arginine is also present in the CsGABAgrd sequence. The Loop F lysine that was shown to interact with glutamate in *C*. *elegans* GluCl channel [13] is missing from all *C*. *salei* GluCl subunits and was recently shown to be unnecessary for glutamate binding [79].

The nAChα subunit and the CsnAChBP share all the agonist binding residues with both invertebrate and vertebrate nAChα receptors (Fig 6). However, these residues are not present in the two isomers of CsnACh1 where the Loop A tyrosine is replaced with another aromatic, histidine. The nicotine binding tryptophan in Loop D is occupied with a tyrosine that is commonly found in anion selective channels [80]. Based on the lack of Loop C vicinal cysteines that are essential for agonist binding [68], these sequences can be classified as non-α subunits.

#### 4.2. Transmembrane helix 2 (TM2)

Fig 7 uses the standard numbering system for LGIC TM2 domain [43]. Starting from highly conserved residue 0’ close to the intracellular C-terminal end, moving inward through the channel, residues in positions 2’, 6’, 13’ and 16’ line the Cys-loop channel pore and have important roles in controlling channel selectivity and conductance [81]. These residues in CsGABArdl are identical to the *Drosophila* rdl sequence (red residues in Fig 7). Alanine in position 2’ is the residue that, when mutated to serine in *Drosophila* and many other insect subunits, renders the channel resistant to the insecticide dieldrin [82,83]. The same residue is also implicated in binding other drugs, such as the Cl-channel blocker picrotoxin and the antiparasitic ivermectin [4,13,84]. Additional positions for picrotoxin and dieldrin block are threonine in position 6’ [84–86] and leucine in position 9’ [87]. Alanine in position 2’ is present in all four *C*. *salei* GABA subunits, the threonine 6’ and leucine 9’ can be found in most anion permeable subunits, except the CsGluCl2 and 3, while the GluCl1 has only one of these residues (Fig 7). Picrotoxin binds between 2’ alanine and -2’ proline in *C*. *elegans* GluCl channel [13]. The -2’ proline is present in both CsGABArdl and CspHCl subunits as well as in CsNC-LGCl. In electrophysiological experiments, the *C*. *salei* VS-3 neuron responses to both GABA_A_ and GluCl receptor agonists were blocked by picrotoxin, but the latter required a significantly higher concentration [26,27]. Since CsGluCl subunits lack all or most of the above mentioned picrotoxin binding residues, it is likely that the VS-3 neuron glutamate response is mediated by heteromultimeric channels.

Anion selective Cys-loop channel α-subunits typically have a PAR motif (proline-alanine-arginine) in the intracellular region between TM1 and TM2 (locations -2’,-1’,0’ in Fig 7), while other subunits have either the proline or alanine, or both, replaced with a different amino acid [10]. All putative *C*. *salei* anion permeable subunits have positively charged arginine in position 0’. This residue is absolutely conserved in Cl-channels [81]. The complete PAR motif is present in both CsGABArdl and CspHCl subunits, as well as the CsHisCl2 and the CsNC-LGCl subunits. CsGABA_A_β, CsGABAgrd and their orthologues in other species do not have proline in the -2’ position. Proline in this position is typical for anion selective channels [10,43,88]. In all grd subunits, alanine in the -1’ position is also replaced. It is likely that the CsGABA_A_β and grd are subunits of heteropentameric channels, rather than either of them forming homopentameric channels in native invertebrate tissues.

The TM2 regions of all three *C*. *salei* and *S*. *mimosarum* GluCl subunits are different to typical anion selective channels. However, even though the -2’and -1’positions do not have proline or alanine, they are not occupied by negatively charged residues as would be expected for cation selective channels [10,43,54,88]. The GluCl sequences in the two spider species also have different residues occupying the locations that line the channel pore [6’,9’,13’] than most anion selective channels. Anion selective channels typically have the polar threonine in position 13’ while this residue is hydrophobic in cation selective channels [43,54,88]. All *C*. *salei* nACh subunits have a hydrophobic residue in this location (either isoleucine or valine), but it is also hydrophobic in CsGluC1 and 2 (isoleucine and leucine, respectively). Therefore, the spider GluCl subunits are quite different to other arthropod sequences and this may contribute to a variability in ion selectivity and channel kinetics.

The CsnAChα subunit shares all of the amino acids in TM2 helix that line the pore and determine the cation selectivity of nACh channels (Fig 7), but both CsnACh1 isomers have significant differences in this helix. Importantly, negatively charged glutamic acids in positions -1’ and 20’ that serve as selectivity filters in cation selective channels [43,73] are occupied by a positively charged lysine and neutral serine, respectively. Therefore, the roles of these subunits are probably different to typical nACh receptors.

#### 4.3. Binding sites for insecticides and antiparasitic agents

Since spiders are ecologically important natural predators for many insects, it is important to determine if widely used insecticides and antiparasitic agents that target Cys-loop receptors also affect spider receptors. As discussed earlier, all four *C*. *salei* GABA receptors have the three TM2 residues (A2’, T6’ and L9’, Fig 7 and S1 Fig) that have been proposed to be involved in binding the cyclodiene insecticide dieldrin, which is now generally banned due to non-specific toxicity [89]. These same residues are also suggested to be involved in binding phenylpyrazoles such as the widely used slow acting insect poison fipronil [6,87]. An additional residue for fipronil binding is a threonine in the TM3 motif (S1 Fig), which is found in the two *C*. *salei* GABArdl subunits. Based on these similarities, all *C*. *salei* GABA subunits are likely to bind dieldrin while CsGABArdl1 and 2 would also be affected by fipronil. These same residues are also present in *S*. *mimosarum* GABA_A_β1 and β2 subunits, indicating that the same control agents would affect these behaviorally different spiders.

**Fig 8:**
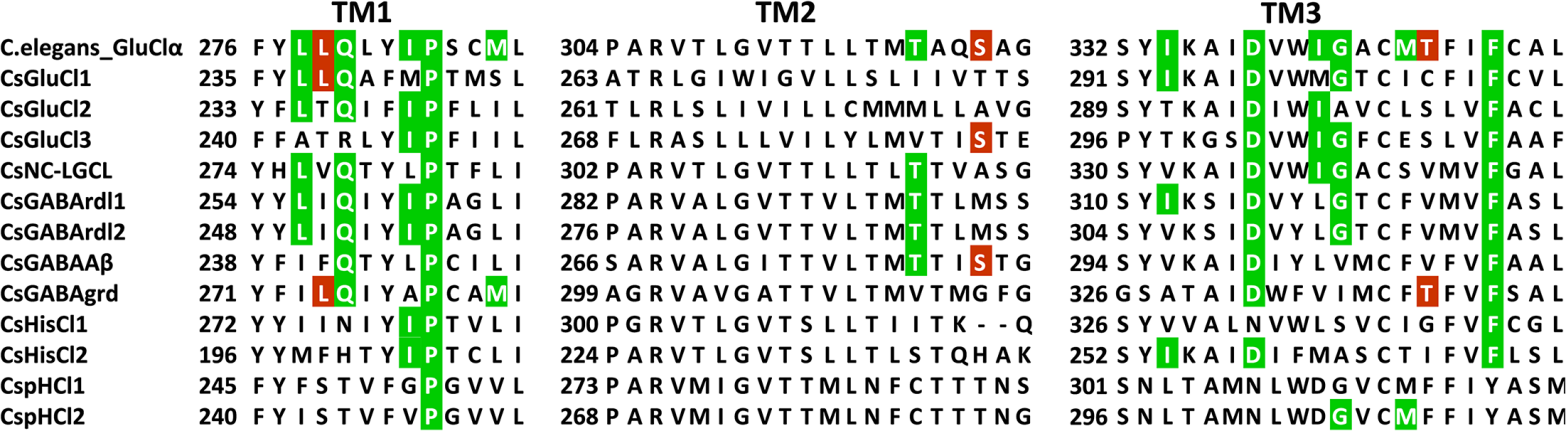
Residues potentially involved in ivermectin binding. Partial alignment of putative *C*. *salei* anion channel subunits with the *C*. *elegans* GluClα sequence. Residues in the *C*. *elegans* sequence that were predicted to be involved in hydrogen-bonding and van der Waals interactions with ivermectin are shown in brown and green respectively [13]. Alignment was done by MAFFT.

Avermectins such as ivermectin and abamectin are antiparasitics that are used against worms causing river blindness in humans, as well as worms and mites in livestock and pets. Abamectins are also used in ant baits and to protect fruit, vegetable and other crops from mites and insects [81]. Avermectins are allosteric modulators directed against GluCl channels; they bind to the receptor transmembrane domain and stabilize the receptor in an open conformation [13]. When tested in electrophysiological experiments, ivermectin depolarized *C*. *salei* VS-3 neurons irreversibly [27], indicating that some receptors in these neurons have binding sites for this drug. No generic amino acid motif has been shown to bind ivermectin in Cys-loop receptors of all species [5]. However, Hibbs and Gouaux [13] predicted three residues to be involved in hydrogen-bonding and 12 additional residues with van der Waals interactions with ivermectin in the *C*. *elegans* GluClα subunit. Of these, the TM3 glycine was later shown to be essential for high ivermectin [90] and abamectin sensitivity [6]. This location is also important in binding new types of insecticides called meta-diamides that are non-competitive antagonists of GABArdl receptors [91]. Fig 8 shows the M1-M3 region of *C*. *elegans* GluClα sequence aligned with the *C*. *salei* anion channel subunits. Many of the residues associated with ivermectin binding are present in several *C*. *salei* subunits and the GABArdl1 and 2, NC-LGCl, and GluCl1 and 3 also have the TM3 glycine, suggesting that these subunits may be involved in ivermectin binding. Although the main targets of ivermectin are GluCl receptors, many other receptors, including GABA, glycine, HisCl and pHCl receptors are modulated by this drug, usually at higher concentrations than GluCl channels [4,81]. If ivermectin binding sites in *C*. *salei* were similar to the *C*. *elegans* GluClα subunit, the two CsGABArdl subunits would be most strongly affected by this drug.

Neonicotinoids, such as the currently most widely used agricultural insecticide Imidacloprid, bind to insect nACh receptors and, since they are not broken down by AChE, cause overstimulation, paralysis and death. Differently to nicotinoids, neonicotinoids cannot cross vertebrate blood brain barriers and they have a negatively charged nitro or cyano group, which is proposed to interact with positively charged amino acids present on insect, but not mammalian nAChRs [2,3,11]. They have not been shown to be toxic for spider mites *T*. *urticae* [6] or wolf spiders *P*. *pseudoannulata*, although the functionally expressed nACh receptors of the latter were sensitive to several neonicotinoids [8]. Although the aromatic residues in the agonist binding region (Fig 7) are highly conserved in different species, there are differences in other residues in this region even within species that are insensitive to neonicotioids [2,6]. The high affinity for neonicotinoids at the insect nAChR has been attributed to their specific binding orientation and different positioning compared to nicotinoids at the vertebrate nAChR [2,3]. Although we do not know the functions or subunit compositions of spider nACh receptors, they are expressed in both the central and peripheral nervous systems (Fig 1), and it is possible that some of them bind neonicotinoids.

### 5. *C*. *salei* Cys-loop receptor homology modelling

Related proteins usually have similar three-dimensional structures and these structures are often evolutionally even more conserved than would be expected from the sequence similarity [92]. We investigated the structural similarity of *C*. *salei* Cys-loop sequences compared to the templates currently available in the Protein Data Bank (PDB) using the I-TASSER server [41]. Each run created five models and the best model based on I-TASSER scores (Table 2) was selected for further analysis. The C-score is in the range of -5 to 2 and indicates the quality of predicted models; higher C-score value signifies a model with more confidence. The TM score indicates the structural similarity between predicted model and the native structure. A TM-score > 0.5 indicates a model of correct topology and a TM-score < 0.17 means random similarity. The results show that the models were of good to excellent quality, with the exception of CsGABAgrd, which had a C-score of -2.27 and TM score of 0.45. Table 2 also shows the main templates that I-TASSER server used to create the models. The templates with highest identity to each *C*. *salei* sequence are indicated in bold.

**Table 2:** The C- and TM scores of *Cupiennius* Cys-loop subunit 3D structures predicted by I-TASSER server, the Protein data bank (PDB) templates used by I-TASSER to create the structures and percentages of residues in favored, allowed and outlier regions as evaluated by Ramachandran plot analysis.

Subunit	C-score	TM score	PDB Templates	#of residues in favored region	#of residues in allowed region	#of residues in outlier region
CsGABArdl1	-1.45	0.54±0.15	**4cof**,4pir,4aq5	81.4%	11.6%	7.0%
CsGABArdl2	-1.12	0.57±0.14	**4cof**	84.4%	9.1%	6.5%
CsGABA_A_β	-1.33	0.55±0.15	**4cof**,4aq5,4pir	88.3%	7.7%	4.0%
CsGABAgrd	-2.27	0.45±0.14	**4cof**,3eam,4aq5,4pir	82.6%	12.5%	4.9%
CsGluCl1	-0.37	0.67±0.13	**4tnw**,**3rhw**,3eam,4cof	88.4%	9.7%	1.9%
CsGluCl2	-0.39	0.66±0.13	**4tnw,3rhw**,4cof,4aq5	86%	9.8%	4.3%
CsGluCl3	-1.10	0.58±0.14	**4tnw,3rhw**,4cof,4hfi	84.3%	10.7%	5%
CspHCl1	-0.53	0.65±0.13	**3rhw**,**4tnw**,4cof,4pir	81.5%	11.4%	7.1%
CspHCl2	-0.59	0.64±0.13	**3rhw**,**4tnw**,4pir,4cof	79.9%	12.9%	7.2%
CsHisCl1	-1.04	0.58±0.14	**3rhw**,**4tnw**,4cof	80.4%	10.6%	9%
CsHisCl2	-0.28	0.68±0.12	**3rhw,** 4cof, 3eam	79.9%	13.8%	6.3%
CsNC-LGCl	-0.80	0.61±0.14	**4tnw,**3rhw,4cof	85.8%	9.1%	5.1%
CsnACh1A	-0.91	0.60±0.14	**4pir**,4aq5,4cof	84.1%	11.4%	4.7%
CsnACh1B	-0.64	0.63±0.13	**4pir**,4aq5,2qc1	84.1%	11.1%	4.8%
CsnAChα	-1.48	0.53±0.15	**2bg9**,4pir,4aq5	75.5%	15.3%	9.2%
CsAChBP	-0.26	0.68±0.12	**3sq6**,4d01,2qc1,4aq5,2bg9	81%	13.7%	5.3%

4cof *H*. *sapiens* GABA_A_β3, 4pir *M*. *musculus* 5-HT_3_; 4aq5 and 2bg9 *T*. *marmorata* nACh receptor, 3eam and 4hfi *G*. *violaceus* GLIC, 3rhw *C*. *elegans* GluClα open channel, 4tnw C. elegans GluClα closed channel, 2qc1 *M*. *musculus* extracellular domain of nAChRα1, 3sq6 *H*. *sapiens* and *L*. *stagnalis* ligand binding domain nAChα7 Chimera, 4d01 *H*. *sapiens* nAChα9.

**Fig 9:**
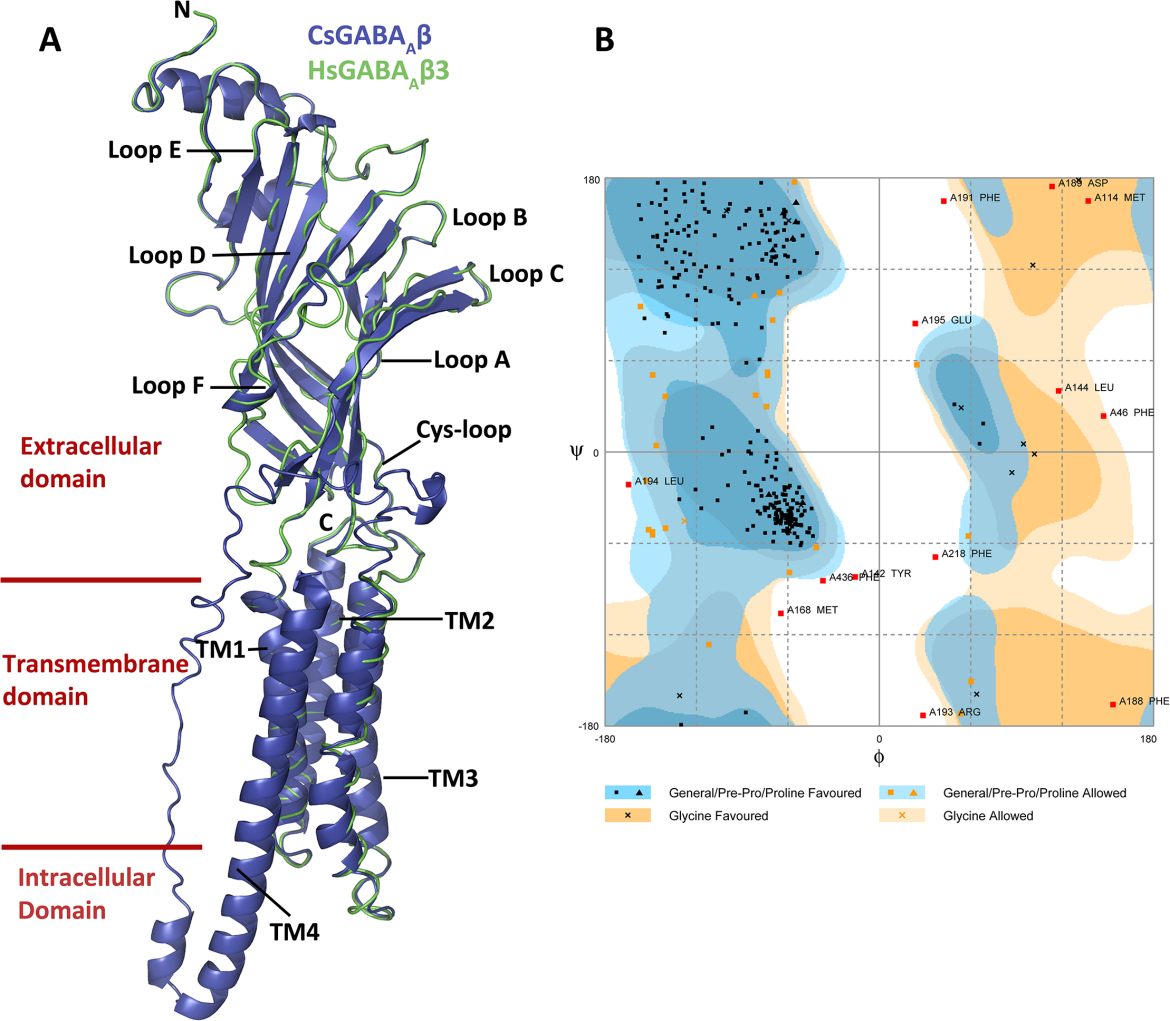
Structural model of the *C*. *salei* GABA_A_β subunit and its evaluation by Ramachandran plot analysis. (A) Homology model of CsGABA_A_β created by I-TASSER server is shown in blue as cartoon representation aligned with the human HsGABA_A_β3 crystal structure in green ribbon representation. The structures are shown as viewed from the outside of the pentameric ring. The agonist binding Loops A-B in the principal face and D-F in the complementary face and the transmembrane helices TM1-M4 are indicated. Image was created with PYMOL software. (B) Ramachandran plot analysis of CsGABA_A_β protein structure calculated by the Rampage server.

A predicted cartoon model for CsGABA_A_β (Fig 9) is aligned with a ribbon representation of the human GABA_A_β subunit (4cof). Similar images from all *C*. *salei* sequences aligned to the templates with highest identities are shown in S3 Fig. All models have the large, β-sheet-rich, extracellular domain with ligand-binding loops and an α-helical pore-forming domain. The intracellular domain, which is believed to interact with cellular scaffolding proteins in most Cys-loop receptors, is present in all but the three CsGluCl and the CsNC-LGCL models. This intracellular domain is also missing in the *C*. *elegans* GluClα crystal structure [13].

All three *C*. *salei* GABA subunit models shared highest identity (43–59%) with the human GABA_A_β3 crystal structure [15]. *C*. *salei* models were well aligned with this structure, except in the N-terminal ligand binding region of CsGABAgrd. All other putative anion channel subunits shared highest identity (31–44%) with the *C*. *elegans* GluCl crystal structure [14,15]. The CsGluCl1-3 and CsNC-LGCl models were well aligned with this model, but CspHCl1 and 2 as well as CsHisCl1 and 2 N-terminal extracellular domains did not match as well. Interestingly, the two isomers of *C*. *salei* nACh1 subunits were best aligned with the mouse 5-HT_3_ crystal structure [16] rather than any of the available nACh structures. Even though the sequence identity of these proteins was only 23%, their ligand binding extracellular regions and TM regions were well aligned. Extracellular regions of nAChα and AChBP sequences have all the basic structures predicted to be required for ligand binding, but they were poorly aligned with any available models. They share relatively high sequence identity with several nACh receptor crystal structures, but most of these are rather low resolution models.

We evaluated the overall stereochemical qualities of each homology model with Ramachandran plots using the RAMPAGE server [42]. The highly variable segment between TM3 and TM4 is not included in several of the Protein data bank templates that I-TASSER used as models. These segments are clearly visible in many of the *C*. *salei* models and not aligned with their templates (Fig 9, S3 Fig). Therefore, these segments were removed before the models were submitted to the Rampage server. An example of a Ramachandran plot is shown in Fig 9 and the overall results are listed in Table 2. The results indicated that the predicted three-dimensional models are of good quality. All models had some residues in the outlier region, indicating that the combination of phi and psi torsion angles were unusual, but these were mainly in regions distant from the binding site or channel pore, close to the C- or N-terminal regions or in the region between the extracellular ligand-binding and transmembrane segments. Only two outlier residues were in transmembrane helices; in models CsGABArdl2 and CspHCl1, in both cases a highly conserved proline in TM1 was indicated to be an outlier. Most residues in the extracellular agonist binding loops were in the favoured or allowed regions, but 1–3 outliers were present in loop C and also in the coil region close to loop C. The only sequences without these outliers were CsGluCl1 and CspHCl1 and 2. It is not clear if these outliers are truly unusual angles in the protein folding in these functionally important regions, or model errors. Only a small number of high-resolution Cys-loop protein models are available in PDB and even fewer have been crystallized in more than one state (open and closed). From the agonist binding loops, Loop C undergoes most pronounced movement when the agonist or antagonist binds to the receptor [1], making it more difficult to predict homology models from this area.

## Conclusions

*C*. *salei* transcriptome searches revealed a relatively small number of Cys-loop receptor subunits compared to available insect or arachnid genomes that have more than 20 such subunits [6] or *C*. *elegans* with 102 Cys-loop subunits [9]. It is likely that additional subunits are expressed in *C*. *salei* organs and tissues that were not included in the transcriptomes. It is also possible that we missed very low abundance genes in the brain and hypodermis. However, we found representatives of all major groups of invertebrate Cys-loop receptors, in some cases more than one orthologous subunit, indicating that these proteins have important roles in the spider central and peripheral nervous systems.

Phylogenetic analysis showed that although the same Cys-loop receptor clades are found in most arthropods, some arachnid clades are distant to insects and some subclades had only spider genes. Unfortunately, there is little molecular, and even less physiological, information currently available about Cys-loop receptors in other spider species. Several *S*. *mimosarum* Cys-loop gene fragments have been submitted to the GeneBank [93] and annotated based on homology, but their functions are unknown. Our analysis suggests that some annotated functions of *S*. *mimosarum*, and other arachnid subunits may not be accurate. It is interesting that even though *C*. *salei* and *S*. *mimosarum* are members of different Araneomorhae families (Lycosoidea and Eresidae, respectively) their Cys-loop receptor genes are closely related. This includes the putative AChBP that in our analysis had both the start and stop codons, indicating that it encodes a complete protein, while *S*. *mimosarum* has a gene fragment in the same clade but we do not know if it also encodes AChBP rather than the complete nACh receptor subunit as annotated.

Although subunit compositions of native invertebrate Cys-loop receptors are unknown, expression of some genes form homomultimeric receptors. Examples are the GABArdl, GluClα and pHCl subunits of several insects [50,52,60,63,64]. However, evidence from several arthropod species indicate that multiple pharmacologically different receptors exist, suggesting that many native Cys-loop receptors are heteromultimeric [59,62]. Examples of these are the invertebrate and most mammalian nACh subunits that alone do not produce functional nACh receptors, but require at least two different subunits [94,95]. When two wolf spider (*P*. *pseudoannulata*) nAChα subunits were each co-expressed with the nAChβ subunit, both formed functional ACh receptors, but not when they were expressed alone [8]. We did not find any *C*. *salei* subunits that would be orthologous to the *P*. *pseudoannulata* nAChβ, but we found two isomers of non-α nACh subunits that may be needed to form functional receptors.

Our sequence analysis and homology models are helpful in functional annotation of the *C*. *salei* Cys-loop receptors. They indicated that many features are similar to currently available templates and suggest that they have similar abilities to bind ligands and drugs, some likely as harmful to spiders as to insect pests and parasites. However, we also found differences that probably lead to different binding properties as well as differences in ion channel kinetics and selectivity. It would be important to express the genes functionally to determine properties of each receptor. Control agent development generally considers safety for humans and other mammals, but little effort has been placed on potential harm to beneficial species such as spiders.

There is significant physiological information about the anion selective Cys-loop receptors that modulate the sensitivity of VS-3 mechanoreceptor neurons innervating slit sensilla in the spider patella [26–29]. We now have molecular tools to identify the proteins that form each of these receptors and determine the roles they play in regulating the flow of information from the spider’s environment to its central nervous system.

## Supporting Information

S1 Fig MAFFT alignment of putative *C*. *salei* anion selective Cys-loop channel subunit amino acid sequences with other Cys-loop receptors. Signal peptides for *C*. *salei* sequences are indicated in red. Secondary structure based on I-Tasser prediction with high confidence (score over 8) is presented for the CsGABArdl1 protein. β-sheets are indicated in green and α-helices in red. Blue lines indicate amino acid sequences that form agonist-binding loops A to G. Cys-loops are identified in brown. TM1-TM4 are the transmembrane segments. Residues potentially involved in dieldrin and fipronil binding in TM2 and TM3 are circled in green. Gray color indicates conserved amino acids based on Jalview consensus threshold of 50% where darker gray indicates higher degree of conservation. Accession numbers for *C*. *salei* sequences are in Table 1 and for other species in the S1 Table.
(PDF)

S2 Fig MAFFT aligned putative *C*. *salei* nACh channel subunits with homologous subunits from other species. Signal peptides for all *C*. *salei* sequences are shown in red. Secondary structure as predicted by I-Tasser for CsnAChα protein shows β-sheets in green and α-helices in red. I-Tasser confidence score was over 8 for these predictions. Amino acid sequences that form agonist-binding loops A to F are indicated as blue lines. Cys-loop and the double cysteine are shown in brown. TM1-TM4 are the transmembrane segments. Gray color indicates conserved amino acids based on Jalview consensus threshold of 50% where darker gray means higher conservation. Accession numbers for *C*. *salei* sequences are in Table 1 and for the other species in S1 Table.
(PDF)

S3 Fig I-Tasser predicted structures for *C*. *salei* Cys-loop subunits. *C*. *salei* structural models are shown in blue cartoon representations aligned with their PDB templates in ribbon representations. Human GABA_A_β3 (HsGABA_A_β3, green 4cof), *C*. *elegans* GluClα (CeGluCl, red 3rhw), mouse 5-HT3 (Mm5-HT3, orange 4pir), *Torpedo marmorata* nAChα (TmnAChα, cyan 4aq5) and *Lymnae stagnalis*/Human nAChα7 ligand binding domain chimera (LsHsnAChα7, cyan 3sq6). Images were created with PYMOL software.
(PDF)

S1 Table List of genes used for analysis. (PDF)
