# Supplementary material for: Transcriptome Analysis of the Central and Peripheral Nervous Systems of the Spider Cupiennius salei Reveals Multiple Putative Cys-Loop Ligand Gated Ion Channel Subunits and an Acetylcholine Binding Protein
Source: PLoS One. 2015 Sep 14;10(9):e0138068. doi: 10.1371/journal.pone.0138068 (PMC4569296; doi:10.1371/journal.pone.0138068)
Supplement: S2 Fig — Signal peptides for all C. salei sequences are shown in red. Secondary structure as predicted by I-Tasser for CsnAChα protein shows β-sheets in green and α-helices in red. I-Tasser confidence score was over 8 for these predictions. Amino acid sequences that form agonist-binding loops A to F are indicated as blue lines. Cys-loop and the double cysteine are shown in brown. TM1-TM4 are the transmembrane segments. Gray color indicates conserved amino acids based on Jalview consensus threshold of 50% where darker gray means higher conservation. Accession numbers for C. salei sequences are in Table 1 and for the other species in S1 Table. (PDF) [file pone.0138068.s002.pdf]

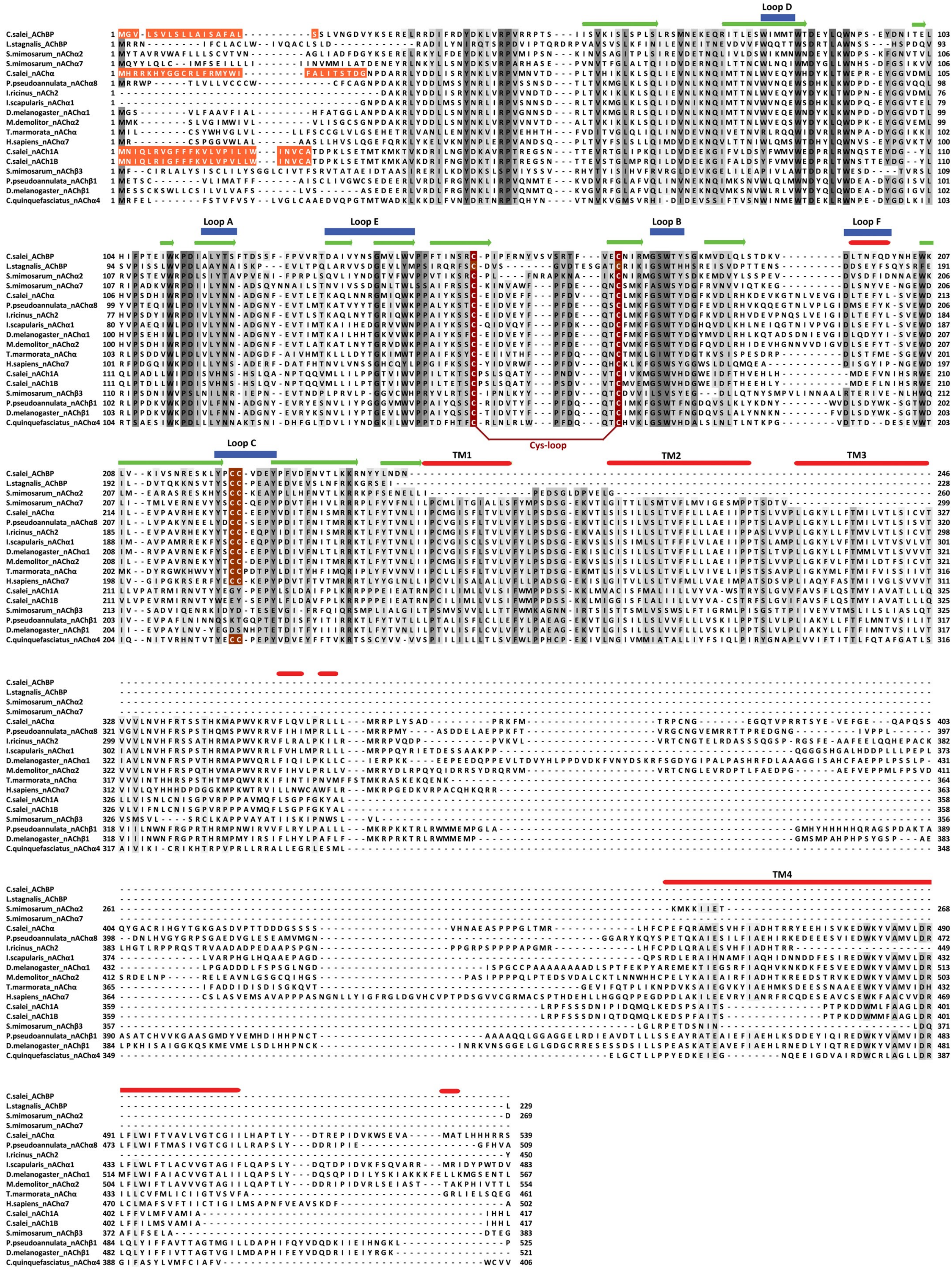
Loop D
Loop A
Loop E
Loop B
Loop F
Loop C
Cys-loop
TM1
TM2
TM3
TM4
C.salei_AChBP
L.stagnalis_AChBP
S.mimosarum_nACha2
S.mimosarum_nACha7
C.salei_nACha
P.pseudoannulata_nACha8
I.ricinus_nACh2
I.scapularis_nACha1
D.melanogaster_nACha1
M.demolitor_nACha2
T.marmorata_nACha
H.sapiens_nACha7
C.salei_nACh1A
C.salei_nACh1B
S.mimosarum_nAChβ3
P.pseudoannulata_nAChβ1
D.melanogaster_nAChβ1
C.quinquefasciatus_nACha4
