# Supplementary figures and images for: Transcriptome Analysis of the Central and Peripheral Nervous Systems of the Spider Cupiennius salei Reveals Multiple Putative Cys-Loop Ligand Gated Ion Channel Subunits and an Acetylcholine Binding Protein
Source: PLoS One. 2015 Sep 14;10(9):e0138068. doi: 10.1371/journal.pone.0138068 (PMC4569296; doi:10.1371/journal.pone.0138068)

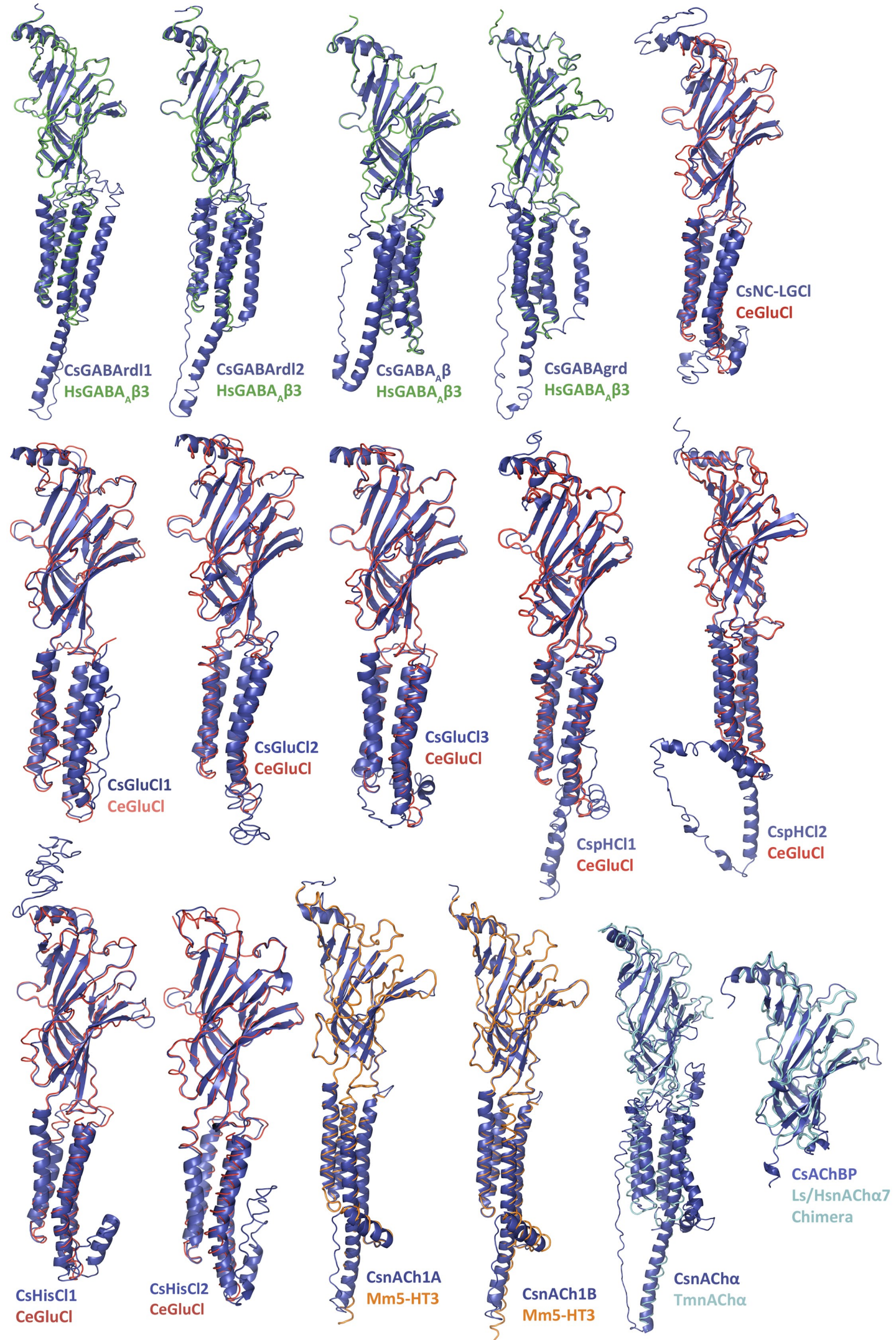

Supplement: S3 Fig — C. salei structural models are shown in blue cartoon representations aligned with their PDB templates in ribbon representations. Human GABAAβ3 (HsGABAAβ3, green 4cof), C. elegans GluClα (CeGluCl, red 3rhw), mouse 5-HT3 (Mm5-HT3, orange 4pir), Torpedo marmorata nAChα (TmnAChα, cyan 4aq5) and Lymnae stagnalis/Human nAChα7 ligand binding domain chimera (LsHsnAChα7, cyan 3sq6). Images were created with PYMOL software. (PDF) [file pone.0138068.s003.pdf]
