# Supplementary material for: Transcriptome Analysis of the Central and Peripheral Nervous Systems of the Spider Cupiennius salei Reveals Multiple Putative Cys-Loop Ligand Gated Ion Channel Subunits and an Acetylcholine Binding Protein
Source: PLoS One. 2015 Sep 14;10(9):e0138068. doi: 10.1371/journal.pone.0138068 (PMC4569296; doi:10.1371/journal.pone.0138068)
Supplement: S1 Table — (PDF) [file pone.0138068.s004.pdf]

S1 Table: List of genes used for analysis

Phylum/class	Species	Protein	Uniprot / *NCBI code*	PDB
**Arthropoda/insecta**	*Acyrthosiphon pisum*	NC (comp)	J9JTU7	
	Aedis aegypti	GABArdl (comp) Ho	Q16896	
	Anopheles gambiae	nAChRα9 (comp)	Q7PSA8	
	Apis mellifera	pHCl (comp)	A0A088AFF8	
		GABAlcch3 (comp)	A0A088A2R0	
		GABAgrd (comp)	A0A087ZPX8	
	Bombyx mori	GluCl3b (comp)	V9P9S1	
		GABAgrd (comp)	E0X9H5	
	Culex quinquefasciatus	nAChRα4 (comp)	B0W5Q9	
	Drosophila melanogaster	nAChα1 (comp) He	P09478	
		nAChβ1 (comp) He	P04755	
		GluClα (comp) Ho	Q94900	
		pHClA (frag) Ho	Q5D6W1	
		HisCl1 (comp) Ho	Q9VGI0	
		HisCl2 (comp) (ort) Ho	A1KYB3	
		GABArdl (comp) Ho/He	P25123	
		GABAlcch3 (comp) He	Q08832	
		GABAgrd (comp) He	Q24352	
	Locusta migratoria	nAChα8 (comp)	W6EAG0	
	Microplitis demolitor	nAChα1 (comp)	*XP_008553229*	
		nAChα2 (comp)	*XP_008549166*	
		nAChα7 (comp)	*XP_008560787*	
	Musca domestica	GABArdl (comp) Ho	Q75NA5	
	Nasonia vitripennis	nAChα11 (comp)	D3UA23	
		GluCl (comp)	D3UAF9	
		pHCl (comp)	D3UAF2	
		HisCl1 (comp)	D3UAF0	
		GABAlcch3 (comp)	D3UAF7	
	Tribolium castaneum	nAChα10 (comp)	A8DIV1	
		pHCl (comp)	A8DMU9	
		HisCl1 (comp)	A8DMU7	
		HisCl2 (comp)	A8DMU8	
		GABAlcch3 (comp)	A8DMU3	
		GABAgrd (comp)	A8DMU2	
**Arthoropda/arachnida**	*Dermacentor variabilis*	GluCl10-2 Ho	US patent 7267964	
		GluCl1-GluCl11 Ho	US patent EP2009021	
	Ixodes ricinus	nACh1 (comp)	V5H4U2	
		nACh2 (frag)	V5HYY9	
	Ixodes scapularis	nAChα1 (frag)	A0A088BD75	
		GluCl1 (frag)	R9S2C9	
		GluCl2 (frag)	R9S0M9	
		GluCl3 (frag)	B7Q8K2	
		Glyα1 (frag)	B7P8W0	
		HisCl (frag)	R9S1B2	
		$GABA_A$α (comp)	B7PLP1	

Phylum/class	Species	Protein	Uniprot / *NCBI code*	PDB
		$GABA_A$1 (comp)	B7Q4M3	
		$GABA_A$2 (frag)	R9S1A7	
		$GABA_A$3 (frag)	B7Q4M8	
	Metaseiulus occidentalis	nAChα1A (comp)	*XP_003745665*	
		nAChα1B (comp)	*XP_003738108*	
		nAChα1C (comp)	*XP_003738107*	
		Glyα2 (comp)	*XP_003748161*	
		$GABA_A$α (comp)	*XP_003747426*	
		$GABA_A$β (comp)	*XP_003747411*	
	Pardosa pseudoannulata	nAChα1 (comp) He	D6QYZ4	
		nAChα8 (comp) He	D6QYZ5	
		nAChβ1 (comp) He	C7EA46	
	Rhipicephalus microplus	$GABA_A$ (comp)	C8CGT5	
	Rhipicephalus pulchellus	nACh (frag)	L7MKP0	
		$GABA_A$ (comp)	L7LVP4	
		Glyα3 (comp)	L7M4K6	
	Rhipicephalus sanguineus	nAChα1 (comp) He	V9Z9T0	
	Sarcoptes scabiei	pHCl (frag) Ho	A8SDR1	
	Stegodyphus mimosarum	nAChα2 (frag)	A0A087UEE3	
		nAChα7 (frag)	A0A087U1S9	
		nAChα10 (frag)	A0A087U0W4	
		nAChβ3 (frag)	A0A087TJD1	
		$GABA_A$α (frag)	A0A087TN57	
		$GABA_A$β1 (frag)	A0A087TN58	
		$GABA_A$β2 (frag)	A0A087TN61	
		GluCl1 (frag)	A0A087TG26	
		GluCl2 (frag)	A0A087UMK3	
		GluCl3 (frag)	A0A087UMK5	
		GluCl4 (frag)	A0A087UMK6	
		GlyαZ1 (frag)	A0A087UNJ7	
		Glyα2 (frag)	A0A087UNJ8	
	Tetranychus urticae	nAChα3 (comp)	T1JSJ8	
		nAChα7 (comp)	T1KYX4	
		nAChβ1 (comp)	T1KCW6	
		GluCl1 (frag)	H9U2V4	
		HisCl1 (comp)	T1KWX0	
		pHCl (comp)	T1K9D2	
		GABArdl2 (comp)	T1L3N8	
**Arthropoda/Chilopoda**	*Strigamia maritima*	NC2 (comp)	T1J0D0	
**Arthropoda/crustacea**	*Daphnia pulex*	NC1 (comp)	E9HDH4	
		NC2 (comp)	E9H343	
**Nematoda/Chromadorea**	*Caenorhabditis elegans*	nAChβ3 (comp) He	Q93149	
		GluClα (comp)<br>Ho/He/B	G5EBR3	3rhw<br>4tnw
**Annelida**	*Capitella teleta*	AChBP (comp) B	I6L8L2	
**Mollusca/gastropoda**	*Lymnaea stagnalis*	$GABA_A$β (comp) He	P26714	
		AChBP (com) B	P58154	1i9B
	Sepia officinalis	$GABA_A$β (comp)	Q9GYU4	
**Chordata/elasmobranchii**	*Torpedo marmorata*	nAChα (comp) B	P02711	2bg9

Phylum/class	Species	Protein	Uniprot / *NCBI code*	PDB
**Chordata/mammalia**	*Homo sapiens*	nAChα7 (comp) Ho	P36544	
		nAChα9 (comp) He	Q9UGM1	4do1
		nAChβ3 (comp) No	Q05901	
		$GABA_A$α2 (comp)	P47869	
		$GABA_A$β3 (comp) Ho	P28472	4cof
		Glyα1 (comp) Ho	P23415	
		Glyα2 (comp) Ho	P23416	
	Mus Musculus	$5HT_3$A Ho	P23979	4Pir

NC = uncharacterized, “comp” indicates complete genes and “frag” gene fragments. NCBI codes are shown in italics for sequences where Uniprot codes are not available. PDB; protein data bank code.

Ho = formed homomultimeric channels in expression systems, He= heteromultimeric channels in expression system, B= ligand binding tested. lcch3 = ligand gated chloride ion channel homolog 3. rdl = resistance to dieldrin. grd = GABA and glycine-like receptor.
