# Supplementary material for: Transcriptome Analysis of the Central and Peripheral Nervous Systems of the Spider Cupiennius salei Reveals Multiple Putative Cys-Loop Ligand Gated Ion Channel Subunits and an Acetylcholine Binding Protein
Source: PLoS One. 2015 Sep 14;10(9):e0138068. doi: 10.1371/journal.pone.0138068 (PMC4569296; doi:10.1371/journal.pone.0138068)
Supplement: S1 Fig — Signal peptides for C. salei sequences are indicated in red. Secondary structure based on I-Tasser prediction with high confidence (score over 8) is presented for the CsGABArdl1 protein. β-sheets are indicated in green and α-helices in red. Blue lines indicate amino acid sequences that form agonist-binding loops A to G. Cys-loops are identified in brown. TM1-TM4 are the transmembrane segments. Residues potentially involved in dieldrin and fipronil binding in TM2 and TM3 are circled in green. Gray color indicates conserved amino acids based on Jalview consensus threshold of 50% where darker gray indicates higher degree of conservation. Accession numbers for C. salei sequences are in Table 1 and for other species in the S1 Table. (PDF) [file pone.0138068.s001.pdf]

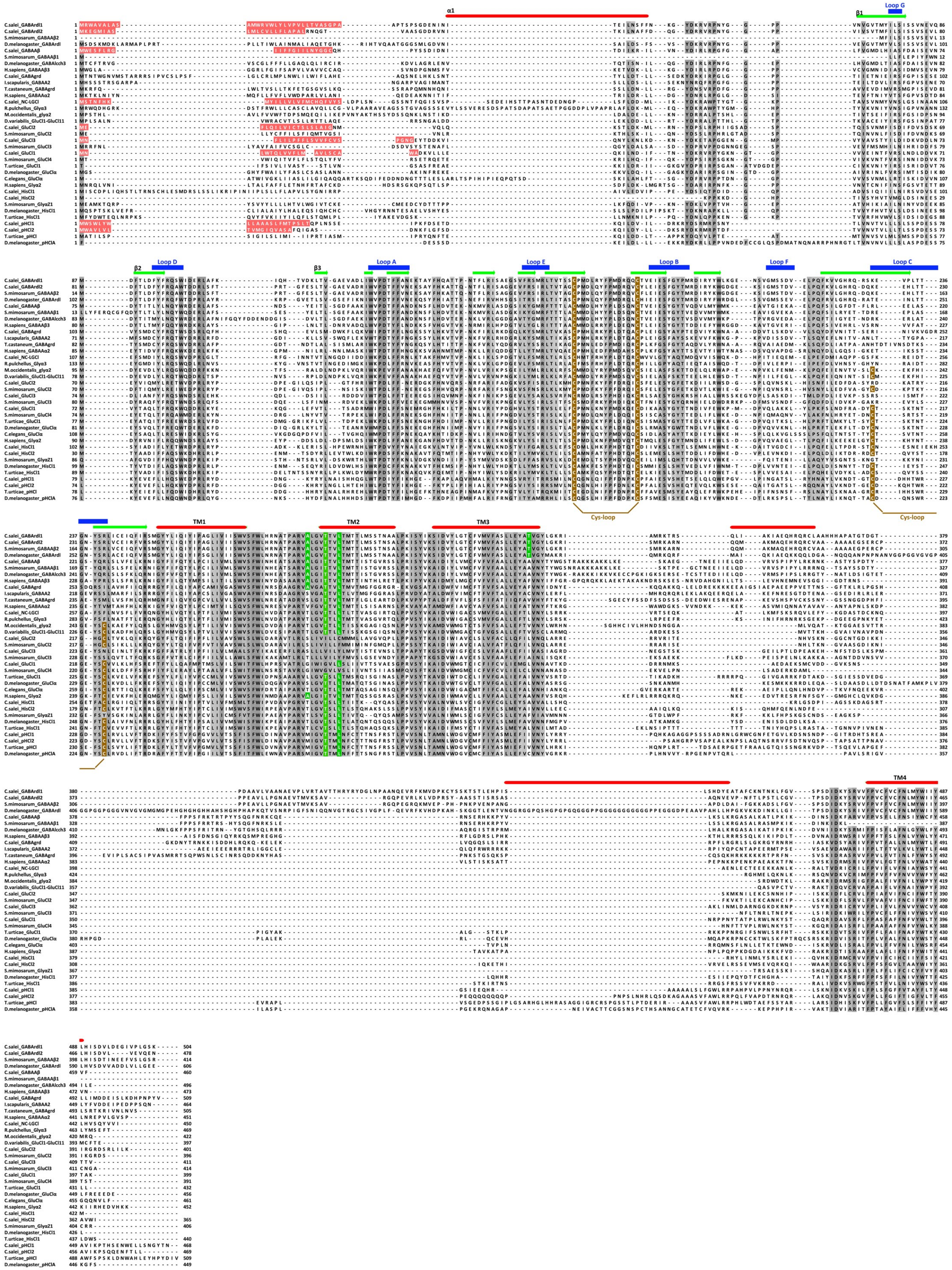
α1
Loop G
β1
β2
Loop D
β3
Loop A
Loop E
Loop B
Loop F
Loop C
Cys-loop
Cys-loop
TM1
TM2
TM3
TM4
C.salei_GABArdl1
C.salei_GABArdl2
S.mimosarum_GABAAβ2
D.melanogaster_GABArdl
C.salei_GABAAβ
S.mimosarum_GABAAβ1
D.melanogaster_GABAlcch3
H.sapiens_GABAAβ3
C.salei_GABAgrd
I.scapularis_GABAA2
T.castaneum_GABAgrd
H.sapiens_GABAAα2
C.salei_NC-LGCl
R.pulchellus_Glyα3
M.occidentalis_glyα2
D.variabilis_GluCl1-GluCl11
C.salei_GluCl2
S.mimosarum_GluCl2
C.salei_GluCl3
S.mimosarum_GluCl3
C.salei_GluCl1
S.mimosarum_GluCl4
T.urticae_GluCl1
D.melanogaster_GluClα
C.elegans_GluClα
H.sapiens_Glyα2
C.salei_HisCl1
C.salei_HisCl2
S.mimosarum_GlyαZ1
D.melanogaster_HisCl1
T.urticae_HisCl1
C.salei_pHCl1
C.salei_pHCl2
T.urticae_pHCl
D.melanogaster_pHClA
